# Roles of Proteins Containing Immunoglobulin-Like Domains in the Conjugation of Bacterial Plasmids

**DOI:** 10.1128/msphere.00978-21

**Published:** 2022-01-05

**Authors:** Mário Hüttener, Jon Hergueta, Manuel Bernabeu, Alejandro Prieto, Sonia Aznar, Susana Merino, Joan Tomás, Antonio Juárez

**Affiliations:** a Department of Genetics, Microbiology and Statistics, University of Barcelona, Barcelona, Spain; b Institute for Bioengineering of Catalonia, The Barcelona Institute of Science and Technology, Barcelona, Spain; University of Iowa

**Keywords:** antimicrobial resistance, bacterial Ig-like proteins, plasmid conjugation

## Abstract

Horizontal transfer of bacterial plasmids generates genetic variability and contributes to the dissemination of the genes that enable bacterial cells to develop antimicrobial resistance (AMR). Several aspects of the conjugative process have long been known, namely, those related to the proteins that participate in the establishment of cell-to-cell contact and to the enzymatic processes associated with the processing of plasmid DNA and its transfer to the recipient cell. In this work, we describe the roles of newly identified proteins that influence the conjugation of several plasmids. Genes encoding high-molecular-weight bacterial proteins that contain one or several immunoglobulin-like domains (Big) are located in the transfer regions of several plasmids that usually harbor AMR determinants. These Big proteins are exported to the external medium and target two extracellular organelles: the flagella and conjugative pili. The plasmid gene-encoded Big proteins facilitate conjugation by reducing cell motility and facilitating cell-to-cell contact by binding both to the flagella and to the conjugative pilus. They use the same export machinery as that used by the conjugative pilus components. In the examples characterized in this paper, these proteins influence conjugation at environmental temperatures (i.e., 25°C). This suggests that they may play relevant roles in the dissemination of plasmids in natural environments. Taking into account that they interact with outer surface organelles, they could be targeted to control the dissemination of different bacterial plasmids carrying AMR determinants.

**IMPORTANCE** Transmission of a plasmid from one bacterial cell to another, in several instances, underlies the dissemination of antimicrobial resistance (AMR) genes. The process requires well-characterized enzymatic machinery that facilitates cell-to-cell contact and the transfer of the plasmid. Our paper identifies novel plasmid gene-encoded high-molecular-weight proteins that contain an immunoglobulin-like domain and are required for plasmid transmission. They are encoded by genes on different groups of plasmids. These proteins are exported outside the cell. They bind to extracellular cell appendages such as the flagella and conjugative pili. Expression of these proteins reduces cell motility and increases the ability of the bacterial cells to transfer the plasmid. These proteins could be targeted with specific antibodies to combat infections caused by AMR microorganisms that harbor these plasmids.

## INTRODUCTION

Bacterial infectious diseases, despite the availability of antibiotics, remain an important public health issue, representing the second leading cause of death worldwide (1). The gradual increase in the resistance rates of several important bacterial pathogens represents a serious threat to public health (2–4). Indeed, multidrug-resistant bacteria are the cause of a slow-growing pandemic. The dissemination of multiple antimicrobial resistance (AMR) genes has been largely attributed to the acquisition of plasmids by horizontal gene transfer (HGT), especially in Gram-negative bacteria (5–7), as well as in Gram-positive bacteria (8). Plasmids can confer resistance to the major classes of antimicrobials (9).

For decades, plasmid incompatibility (10) has been a useful tool for grouping bacterial plasmids. In recent years, other approaches have been considered. Based on phylogenetic analysis of conjugative relaxase, the protein required to initiate plasmid mobilization through conjugation, plasmids can be grouped into different relaxase families (11). Plasmids belonging to the incompatibility group (Inc) HI (MOB_H_ relaxase family) are widespread in *Enterobacteriaceae* and most commonly include genetic elements encoding multiple AMR determinants (12). IncHI plasmids, often >200 kb in size, share a core of approximately 160 kb. The differences in size are due to the presence of distinct insertion elements, including many AMR determinants (13). IncHI-encoded AMR can be present in enterobacteria such as Salmonella, Escherichia coli (14), Klebsiella pneumoniae (15), and Citrobacter freundii (16). Plasmids of the IncHI2 subgroup predominate in antibiotic-resistant Salmonella isolates. In Salmonella enterica serotype Typhi, more than 40% of isolates harbor an IncHI plasmid (17). In recent years, a novel role of IncHI plasmids in AMR spread has been reported. The emergence of Gram-negative bacteria with AMR, especially those producing carbapenemases, led to reintroduction of colistin as a last resort antibiotic for the treatment of severe infections (18). In contrast to its limited clinical use, colistin is widely used in veterinary medicine (19). In the past, colistin resistance was associated only with chromosomal mutations (20). Nevertheless, plasmid-mediated resistance, conferred by the mobilized colistin resistance gene (*mcr-1*), has emerged recently. Since its discovery in 2016 in China (21), *mcr* genes have been detected in animals, food, the human microbiota, and clinical samples in over 30 countries (22–26). IncHI2 plasmids represent 20.5% of the overall plasmids carrying the *mcr-1* gene worldwide but up to 41% in Europe (27). Of special concern is the presence of the *mcr-1* resistance determinant in *Enterobacteriaceae* carrying carbapenem resistance genes, such as *bla*_NDM_ and *bla*_KPC_. The combination of these AMR determinants seriously compromises the treatment of infections caused by pathogenic strains harboring these plasmids (28, 29). An example of this is the recent report of an AMR clone of the highly virulent E. coli ST95 lineage (14). E. coli ST95 clones cause neonatal meningitis and sepsis. They are usually sensitive to several antibiotics. This clone harbors an IncHI2 plasmid that carries, among other factors, genes encoding determinants of resistance to colistin and multiple other antibiotics (including the extended-spectrum beta-lactamase *bla*_CTX-M-1_). The spread of such an AMR ST95 clone could pose a threat to human health worldwide (14).

IncHI plasmid conjugation has a distinctive feature: while optimal conjugation rates are obtained at temperatures found outside the host (30°C and below), conjugative transfer is repressed at temperatures encountered within the host (37°C) (30, 31). The R27 plasmid is the prototype of IncHI1 plasmids. It harbors the Tn*10* transposon, which confers resistance to tetracycline, and has been exhaustively studied. The R27 replication and conjugation determinants are well characterized (32, 33), and its complete nucleotide sequence is available (34). Several open reading frames (ORFs) from the plasmid R27 (66%) do not show similarity to any known ORFs.

IncA/C plasmids belong to the same MOB_H_ relaxase family as IncHI plasmids. They were originally identified in the 1970s among multidrug-resistant Aeromonas hydrophila and *Vibrio* isolates that infected cultured fish (35, 36). Since the 1990s, these plasmids have received increasing interest because of their role in mobilizing AMR in enterobacteria and other Gram-negative microorganisms (37–40). They have an extremely broad host range that includes members of *Beta*-, *Gamma*-, and Deltaproteobacteria (41) and play a relevant role in the global spread of AMR (42, 43). They represented 50% of all plasmids isolated from *bla*_NDM_-producing Klebsiella pneumoniae of clinical origin characterized in a recent study (44).

Proteins containing an immunoglobulin (Ig)-like domain contain several chains of approximately 70 to 100 amino acid residues present in antiparallel β-strands and organized in two β-sheets that are packed against each other in a β-sandwich. The Ig-like domain has been identified in a large number of proteins with diverse biological functions, is widely distributed in nature, and is present in vertebrates, invertebrates, plants, fungi, parasites, bacteria, and viruses (45). Bacterial proteins containing Ig-like domains (Big) exhibit a wide range of functions. They include fimbrial subunits, adhesins, membrane transporters, and several enzymes (as reviewed in reference 46). In a previous report (47), we studied a high-molecular-weight extracellular protein (the RSP protein) that contains a Big domain and plays an essential role in IncHI plasmid conjugation. Among other targets, the RSP protein appears to be associated with flagella, reducing cell motility. Under specific mating conditions, it could be shown that binding of the RSP protein to the flagella influences conjugation (47). In this report, we present novel data about the roles of these plasmid-encoded Big proteins. We show that two Big proteins bind both flagella and the conjugative pilus to favor conjugation of the IncHI1 plasmid R27. Furthermore, we also show that other groups of plasmids such as IncA/C and IncP2 also encode these proteins. We provide evidence for their role in the conjugation of IncA/C plasmids. The role of plasmid-encoded Big proteins in plasmid conjugation is discussed.

## RESULTS

### The RSP protein interacts *in vitro* both with a new R27-encoded Big protein and with a protein involved in plasmid conjugation.

**FIG 1:**
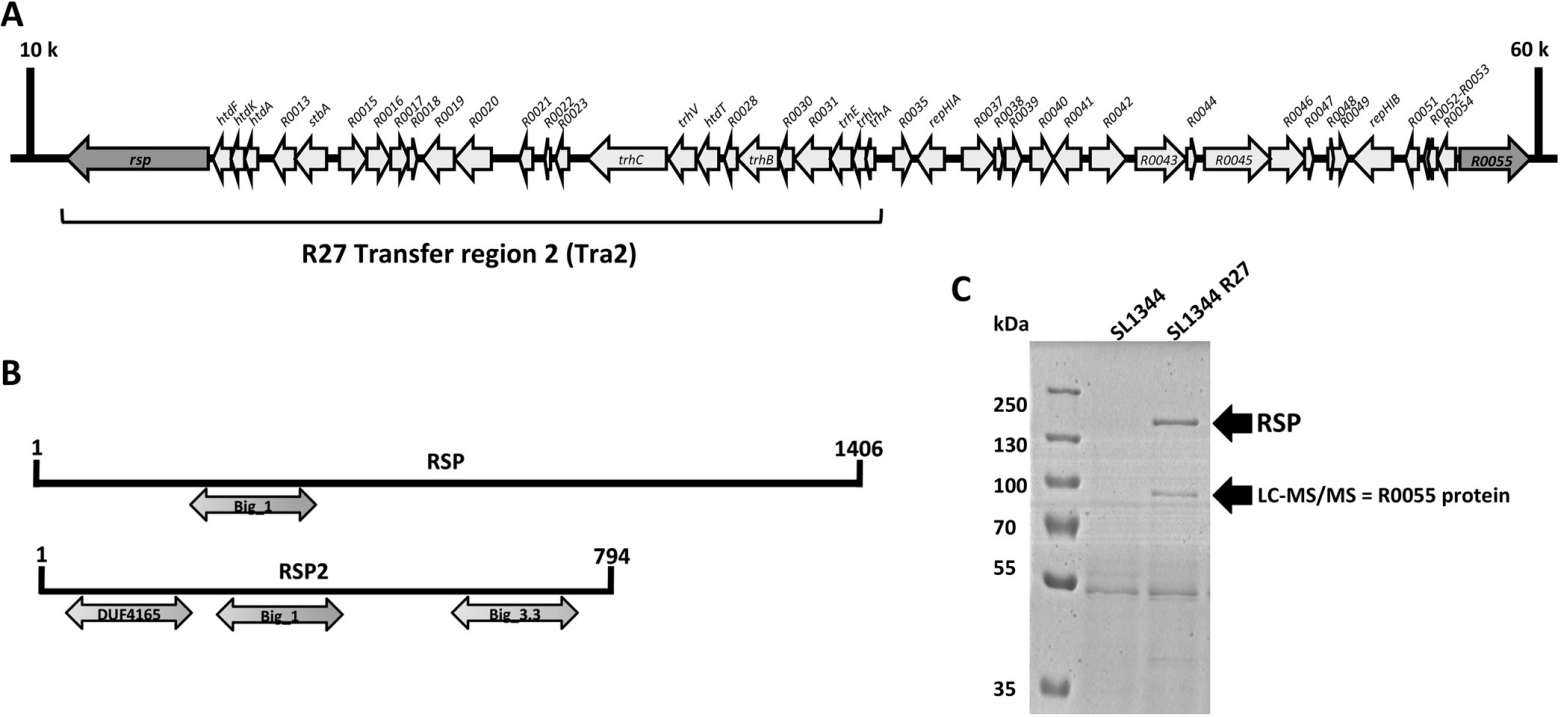
Identification of the RSP2 protein. (A) Genetic map of the R27 plasmid region where both the *rsp* and *rsp2* (*R0055*) genes were mapped (dark gray arrows). (B) Comparison of the Big domains of the RSP and RSP2 proteins. (C) Detection of the RSP2 protein in the cell-free secretome of the SL1344(R27) strain. Arrows point to the bands corresponding to the RSP and RSP2 proteins, the latter of which was confirmed by LC-MS/MS analysis.

To gain further insight into the role of the RSP protein in the conjugation of the R27 plasmid, we decided to assess whether this protein interacts with other proteins expressed by the Salmonella strain SL1344(R27). We performed immunoprecipitation of a cellular extract of strains SL1344(R27 RSP-Flag) and SL1344(R27 Δ*rsp*) and analyzed the proteins that specifically coprecipitated with the RSP protein. Two R27-encoded proteins were found to specifically coprecipitate with the RSP protein (see Table S1 in the supplemental material). The protein showing the highest score (187.77) and coverage (58.56) was the R27_p055 protein. The *R0055* gene was mapped between transfer regions 2 and 1 of the R27 plasmid (Fig. 1A). The *R0055* gene product is a 794-amino-acid (aa) protein with a molecular mass of 86.75 kDa. As with the RSP protein, the protein encoded by *R0055* also contains bacterial Ig-like domains (Big_1 and _3): a Big_1 domain spanning amino acid residues 143 to 254 and a Big_3 domain spanning residues 537 to 693. The R27_p055 protein, herein termed RSP2, also contains a DUF4165 domain of unknown function (amino acids 23 to 142) (Fig. 1B).

10.1128/msphere.00978-21.1 TABLE S1 Proteins copurifying with the RSP protein in an immunoprecipitation experiment. The blue background corresponds to strain SL1344(R27 RSP-Flag). The yellow background corresponds to strain SL1344(R27 Δ*rsp*) (negative control). Download Table S1, XLSX file, 0.01 MB. Copyright © 2022 Hüttener et al. 2022 Hüttener et al. https://creativecommons.org/licenses/by/4.0/ This content is distributed under the terms of the Creative Commons Attribution 4.0 International license.

The second protein showing a high score (59.46) and coverage (39.92) was the R27-encoded TrhH protein. This protein shares 26% of identity with the IncF TraH protein, which is involved in plasmid conjugation (32).

### The RSP2 protein shows RSP-dependent expression in the external medium.

**FIG 2:**
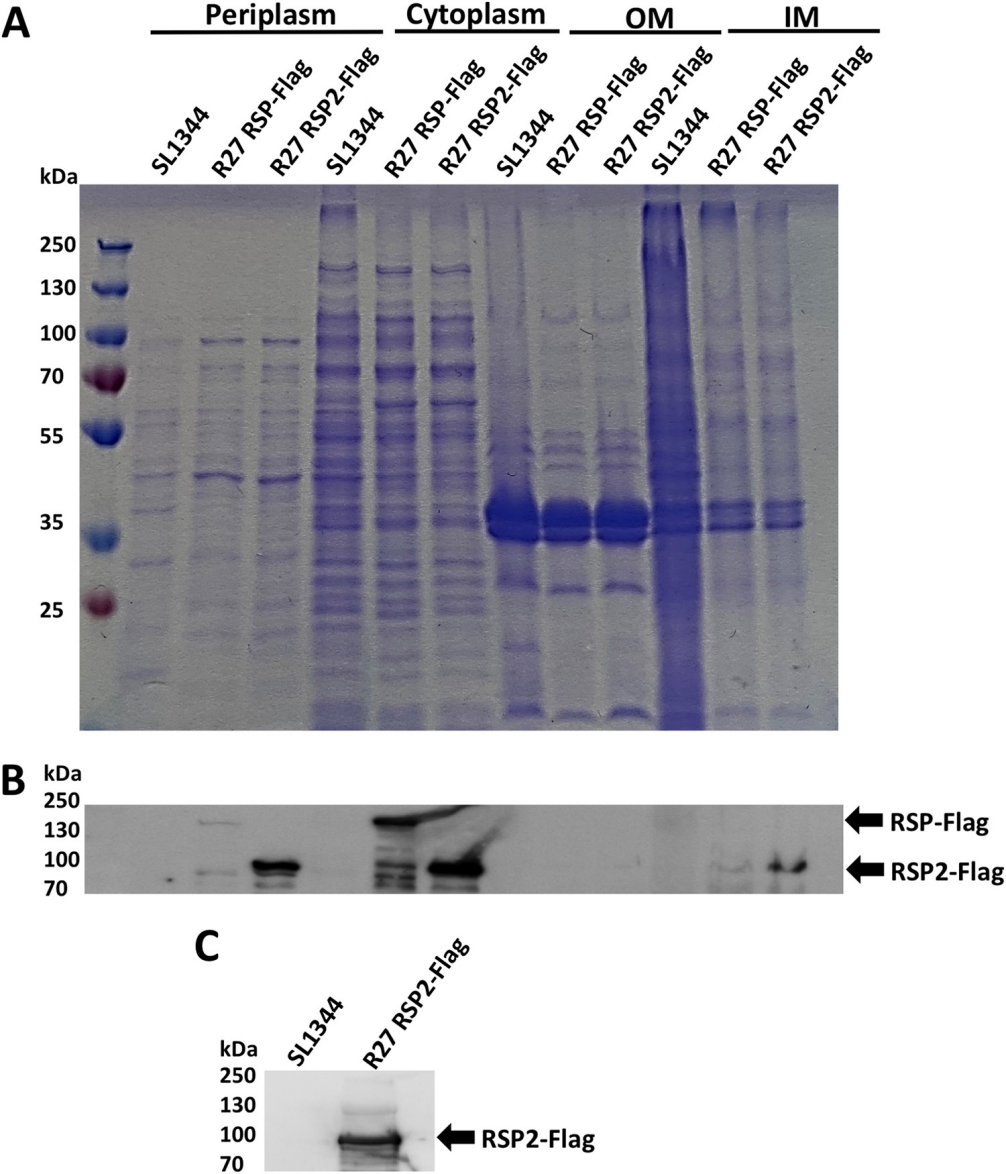
Immunodetection of the RSP2-Flag protein in different cellular compartments. (A) Coomassie blue staining of the different cellular fractions (periplasm, cytoplasm, outer membrane [OM], and inner membrane [IM]) obtained from strains SL11344 wt, SL1344(R27 RSP-Flag) (used as a control), and SL1344(R27 RSP2-Flag). (B) Immunodetection of the RSP-Flag and RSP2-Flag proteins in the periplasm, cytoplasm, and outer and inner membrane fractions of strains SL11344 wt, SL1344(R27 RSP-Flag), and SL1344(R27 RSP2-Flag), respectively. Arrows point to RSP-Flag (control) and RSP2-Flag proteins, respectively. (C) Immunodetection of the RSP2-Flag protein in the cell-free secretome of strains SL11344 wt and SL1344(R27 RSP2-Flag) grown at 25°C until OD_600_ of 2.0. The arrow points to RSP2-Flag protein.

To determine whether the RSP2 protein is also present in the cell-free secretome of strain SL1344(R27), we analyzed the cell-free secreted protein profile of this strain by sodium dodecyl sulfate-polyacrylamide gel electrophoresis (SDS-PAGE) (Fig. 1C). In addition to the band corresponding to the already characterized RSP protein, a second band corresponding to a protein of molecular mass equivalent to that of the RSP2 protein, was observed. The band was isolated and analyzed by liquid chromatography coupled to tandem mass spectrometry (LC-MS/MS). It corresponded to the RSP2 protein (Fig. 1C). To identify the RSP2 protein in the different cellular compartments, a Flag tag was added to the *rsp2* gene (see Materials and Methods for details). Cultures of strains SL1344 wild type (wt) and SL1344(R27 RSP2-Flag) were grown in LB medium at 25°C to an optical density at 600 nm (OD_600_) of 2.0. Samples were then collected, and the different cellular fractions were obtained. The RSP2 protein was detected in the different fractions by Western blotting, using anti-Flag antibodies (Fig. 2A to C). The protein was identified in the same cell compartments as the RSP protein (i.e., periplasm, inner membrane, cytoplasm, and cell-free secreted proteins).

**FIG 3:**
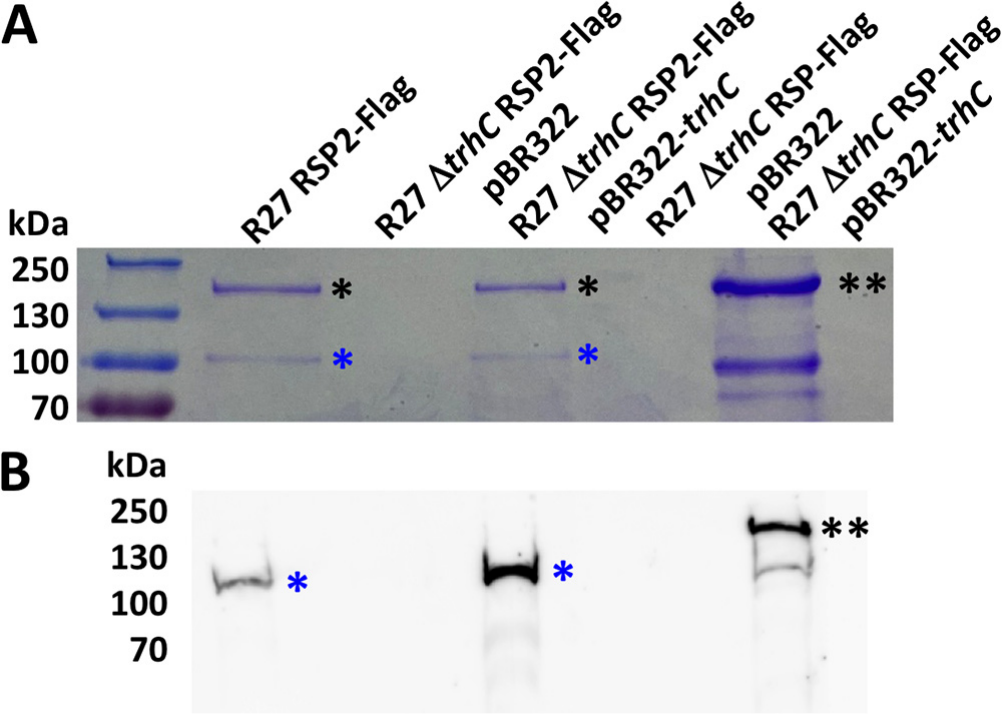
RSP2 export requires the type IV secretion system encoded by the R27 plasmid. (A) Coomassie blue staining for SDS-PAGE analysis of the cell-free secretome of strain SL1344 carrying R27 RSP2-Flag, R27 Δ*trhC* RSP2-Flag pBR322, R27 Δ*trhC* RSP2-Flag pBR322-*trhC*, R27 Δ*trhC* RSP-Flag pBR322, and R27 Δ*trhC* RSP-Flag pBR322-*trhC* grown at 25°C until it reached an OD_600_ of 2.0. A single black asterisk indicates the RSP protein, two black asterisks indicate the RSP-Flag protein (control), and a single blue asterisk indicates the RSP2-Flag protein. The lower band under the RSP-Flag protein must correspond to a degradation variant of RSP-Flag, as it is detected with anti-Flag antibodies. (B) Immunodetection in the cell-free secretome of the RSP-Flag and RSP2-Flag proteins with anti-Flag antibodies. A single blue asterisk indicates the RSP2-Flag protein, and two black asterisks indicate the RSP-Flag protein (control). The experiment was repeated three times. The results of a representative experiment are shown.

As the above reported data show that the RSP2 protein can be exported to the external medium, we decided to study whether this protein is exported by the R27-encoded type IV secretion system that is also used by RSP (47). To address this point, we first used the SSPred program (http://www.bioinformatics.org/sspred/html/sspred.html) for *in silico* prediction of whether the RSP2 protein, similar to the RSP protein, could be exported through a type IV secretion system. To provide evidence supporting this hypothesis, we used strains SL1344(R27 RSP2-Flag) and SL1344(R27 Δ*trhC* RSP2-Flag) and analyzed the presence of RSP2 protein in the protein profile of the cell-free secreted fractions (secretome). This protein could not be detected in the secretome of strain SL1344(R27 Δ*trhC*) (Fig. 3A). Immunodetection of RSP2-Flag by using anti-Flag antibodies confirmed the requirement for TrhC expression for RSP2 export (Fig. 3B). We next checked whether RSP2 export in strain SL1344(R27 Δ*trhC*) could be made to occur by providing the gene encoding the TrhC ATPase in *trans*, cloned in the plasmid pBR322 (plasmid pBR322-*trhC*). Complementation of RSP2 export was observed (Fig. 3B), suggesting that, as was the case for the RSP protein, the R27-encoded type IV secretion system mediates export of the RSP2 protein.

**FIG 4:**
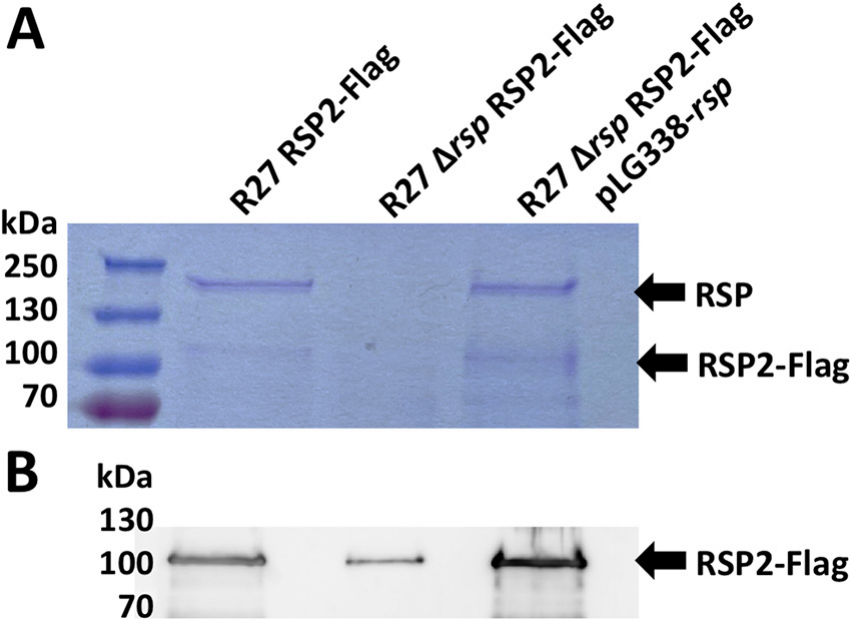
RSP2 export is partially dependent on the expression of the RSP protein. (A) Coomassie blue staining for SDS-PAGE analysis of the cell-free secretomes of the SL1344 strain carrying R27 RSP2-Flag, R27 Δ*rsp* RSP2-Flag, or R27 Δ*rsp* RSP2-Flag pLG338-*rsp* was grown at 25°C until it reached an OD_600_ of 2.0. The arrows point to the RSP and RSP2-Flag proteins. (B) Immunodetection of the RSP2-Flag protein with anti-Flag antibodies in the cell-free secretomes of the strains indicated above. The arrow points to the RSP2-Flag protein. The experiment was repeated three times. The results of a representative experiment are shown.

We next addressed the question whether there is codependence in protein export between the RSP and RSP2 proteins. To that end, a Flag tag was added to the *rsp2* gene in strain SL1344(R27 Δ*rsp*). Expression in the external medium of both RSP and RSP2 proteins was assessed in strains SL1344(R27 RSP2-Flag), SL1344(R27 Δ*rsp* RSP2-Flag), and SL1344(R27 Δ*rsp* RSP2-Flag pLG338-*rsp*) (Fig. 4A and B). The amount of the RSP2 protein is significantly reduced when the RSP protein is not expressed. Hence, the export of the RSP2 protein is at least partially dependent on the RSP expression.

### Expression of the RSP2 protein influences the motility and conjugation of strain SL1344(R27).

We previously showed that the expression of the RSP protein is essential for R27 plasmid conjugation and that SL1344 cells that express RSP show reduced motility compared to plasmid-free cells (47). Considering the observed interaction of both RSP and RSP2 proteins, we studied whether, as is the case for the RSP protein, expression of the RSP2 protein influences cell motility and/or conjugation.

**FIG 5:**
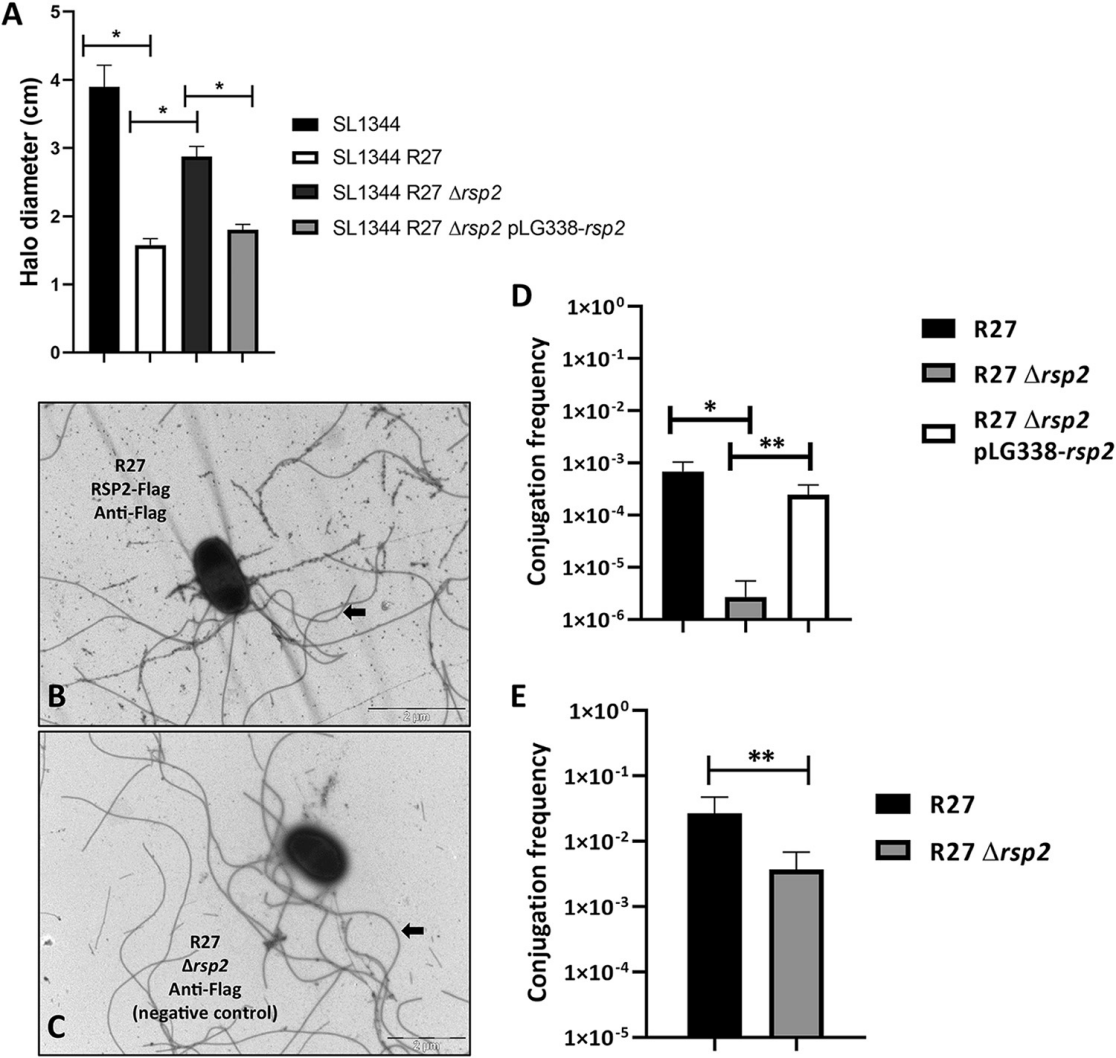
The RSP2 protein influences the motility of strain SL1344(R27) and the conjugation of the R27 plasmid. (A) Effect of the *rsp2* allele on the motility of strain SL1344(R27). The motility of the different strains was measured as the halo diameter of the different colonies growing on motility agar. The results are the means of three independent experiments. Standard deviations are shown by the error bars. Statistical analysis showed significant differences, analyzed by unpaired two-sided Student’s *t* test (*, *P* value < 0.0001). (B and C) The RSP2 protein binds to the flagella of strain SL1344(R27). Immunogold electron microscopy of cells from strain SL1344 carrying R27 RSP2-Flag (B) or SL1344(R27 Δ*rsp2*) (C) using monoclonal anti-Flag antibodies and goat anti-mouse IgG conjugated to 12-nm gold particles. Arrows point to the RSP protein associated with the flagella in panel B and only to the flagella in panel C. Bars represent 2 μm. (D and E) Effect of the *rsp2* allele on the conjugation frequency of the R27 plasmid in liquid (D) and solid (E) media, respectively. The data shown are the means plus standard deviations of three independent experiments. Statistical analysis showed significant differences, analyzed by unpaired two-sided Student’s *t* test (in panel D, *, *P* value = 0.0082; **, *P* value = 0.0095; in panel E, **, *P* value = 0.0428).

After constructing an R27 derivative lacking the *rsp2* gene (plasmid R27 Δ*rsp2*), we performed a comparative motility assay with Salmonella strain SL1344 and its derivatives harboring the R27, R27 Δ*rsp2*, and R27 Δ*rsp2* pLG338-*rsp2* plasmids. The results obtained (Fig. 5A) showed that the RSP2 protein influences the motility of strain SL1344(R27).

We were able to show previously that the RSP protein is associated with the flagella synthesized by Salmonella strain SL1344 (47). We decided next to assess whether RSP2 also targets the flagella. In an attempt to detect the RSP2 protein by transmission electron microscopy, we used strain SL1344(R27 RSP2-Flag) and gold-labeled anti-Flag monoclonal antibodies (Fig. 5B and C). As was the case for RSP, RSP2 also exhibited binding to the flagella.

To assess the role of the RSP2 protein on plasmid conjugation, we constructed a R27 derivative lacking the *rsp2* gene (plasmid R27 Δ*rsp2*) and compared the conjugation frequencies of strains SL1344(R27), SL1344(R27 Δ*rsp2*), and SL1344(R27 Δ*rsp2* pLG338-*rsp2*) growing at 25°C in liquid media. We also compared the conjugation frequencies of strains SL1344(R27) and SL1344(R27 Δ*rsp2*) growing cells on nitrocellulose filters placed on LB plates. When cells were grown in liquid medium, transfer of the R27 Δ*rsp2* plasmid was detected at a frequency that was approximately 2 log units lower than that of wt R27 (Fig. 5D). The presence in *trans* of the RSP2 protein encoded by the pLG338-*rsp2* plasmid restored the conjugation frequency of the wt R27 plasmid. When cells were grown on solid medium, we also detected a significant decrease in the conjugation frequency of the R27 Δ*rsp2* plasmid compared to that of the wt R27 plasmid (Fig. 5E).

### Relationship between the RSP and RSP2 proteins and the conjugative machinery of the R27 plasmid.

**FIG 6:**
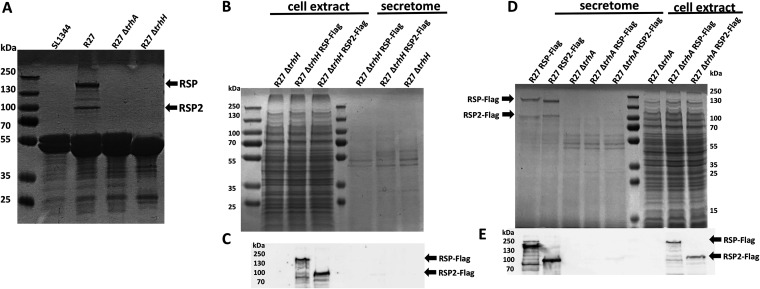
Expression of the RSP and RSP2 proteins depends on the R27 TrhA and TrhH functions. (A) SDS-PAGE analysis of the purified flagellar fractions of the plasmid-free strain SL1344 and strain SL1344 harboring either the R27 plasmid or the R27 Δ*trhA* or R27 Δ*trhH* derivative. Arrows point to the RSP and RSP2 proteins. (B) SDS-PAGE analysis of the cellular extract and cell-free secretome of strain SL1344 carrying R27 Δ*trhH*, R27 Δ*trhH* RSP-Flag, and R27 Δ*trhH* RSP2-Flag, respectively. (C) Immunodetection of the RSP-Flag and RSP2-Flag proteins in the intracellular compartments of strain SL1344 harboring the corresponding *trhH* derivatives of the R27 plasmid. Arrows point to the RSP-Flag and RSP2-Flag proteins. (D) SDS-PAGE analysis of the cellular extract and cell-free secretome of strain SL1344 carrying R27 RSP-Flag, R27 RSP2-Flag, R27 Δ*trhA*, R27 Δ*trhA* RSP-Flag, or R27 Δ*trhA* RSP2-Flag, respectively. Arrows point to the RSP-Flag and RSP2-Flag proteins. (E) Immunodetection of the RSP-Flag and RSP2-Flag proteins in the intracellular compartments of strain SL1344 harboring the corresponding *trhA* derivatives of the R27 plasmid. Arrows point to the RSP-Flag and RSP2-Flag proteins. The experiments were repeated three times. The results of a representative experiment are shown.

**FIG 7:**
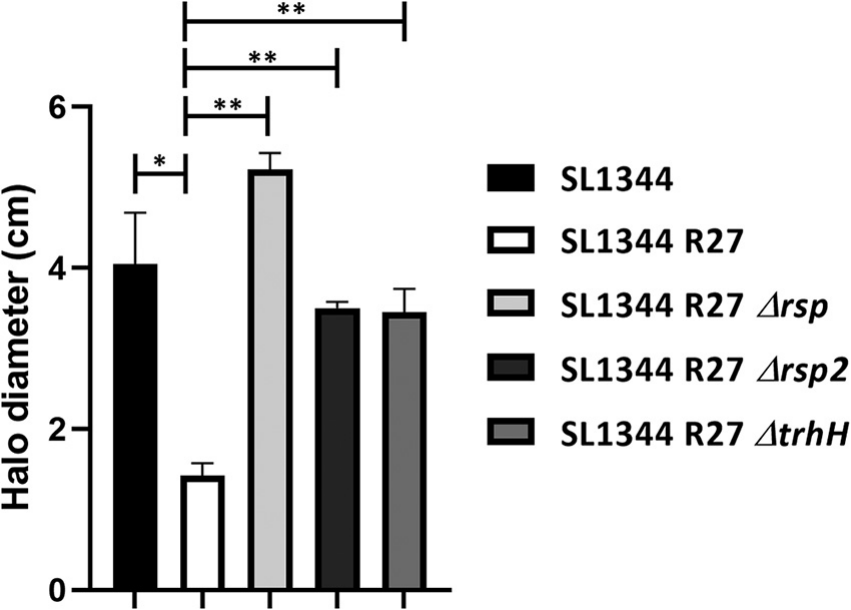
Loss of TrhH function impairs the effect of the RSP and RSP2 proteins on SL1344 cell motility. The motility of the different strains was measured as the halo diameter of the different colonies growing on motility agar. The results are the means of three independent experiments. Standard deviations are shown. The statistical analysis showed a significant difference analyzed by unpaired two-sided Student’s *t* test (*, *P* value = 0.0009; **, *P* value <0.0001).

As mentioned above, immunoprecipitation of the RSP protein indicated interactions with both the RSP protein and the R27 *trhH* gene product. The TrhH protein of plasmid R27 shares identity with the TraH protein encoded by IncF plasmids (32). The TraH protein is a component of the outer membrane complex involved in conjugation (48) and has been shown to be required for pilus assembly (49). On the other hand, the *trhA* gene product encoded in IncHI plasmids has been considered to be the pilin subunit itself (50). We decided to analyze whether expression of the R27 TrhH and/or TrhA proteins encoded by IncHI plasmids influences expression in the external medium of the RSP and RSP2 proteins. After inactivation of the *trhH* or *trhA* gene of strain SL1344(R27), the expression of the RSP and RSP2 proteins in the external medium was analyzed. As both the RSP and RSP2 proteins were copurified with flagella when a conventional flagellar purification protocol was used (see Materials and Methods), we obtained fractions containing flagella from the SL1344 wt, SL1344(R27), SL1344(R27 Δ*trhA*), and SL1344(R27 Δ*trhH*) isogenic derivatives. Proteins were analyzed by SDS-PAGE (Fig. 6A). The expression of both the RSP and RSP2 proteins in the extracellular medium is dependent upon the function of TrhH or TrhA. We also analyzed the intracellular expression of the RSP and RSP2 proteins in the *trhH* and *trhA* genetic backgrounds. Lack of either TrhH (Fig. 6B and C) or TrhA (Fig. 6D and E) function resulted in intracellular accumulation of either RSP or RSP2. The acquisition of the R27 plasmid reduces motility in strain SL1344, and the inactivation of either the *rsp* or *rsp2* gene results in an increased motility of the strain. Therefore, if inactivation of the *trhH* gene interferes with the presence of these proteins in the extracellular medium, it could be expected that *trhH* inactivation would also result in increased motility in SL1344 cells harboring plasmid R27 Δ*trhH*. We then performed a mobility assay of the plasmid-free SL1344 strain and its SL1344(R27), SL1344(R27 Δ*rsp*), SL1344(R27 Δ*rsp2*), and SL1344(R27 Δ*trhH*) derivatives. In accordance with (i) the observed requirement of TrhH function for RSP and RSP2 export and (ii) the effect of the RSP/RSP2 proteins on cell motility, the motility of strain SL1344(R27 Δ*trhH*) was significantly increased compared to that of the SL1344(R27) strain (Fig. 7).

### The RSP and RSP2 proteins bind the R27 conjugative pili.

**FIG 8:**
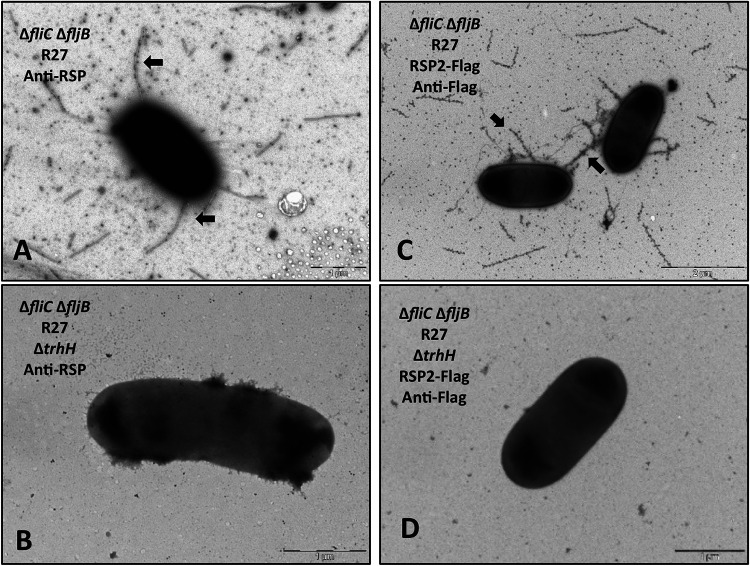
The RSP and RSP2 proteins bind to the conjugative pilus encoded by the R27 plasmid. (A and C) Immunogold electron microscopy of cells from strains SL1344 Δ*fliC* Δ*fljB* (R27) (A) and SL1344 Δ*fliC* Δ*fljB* (R27 Δ*trhH*) (B) using polyclonal anti-RSP antibodies and goat anti-rabbit IgG conjugated to 12-nm gold particles. Arrows point to the RSP protein associated with the conjugative pilus. (C and D) Immunogold electron microscopy of cells from strains SL1344 Δ*fliC* Δ*fljB* (R27 RSP2-Flag) (C) and SL1344 Δ*fliC* Δ*fljB* (R27 RSP2-Flag Δ*trhH*) (D) using anti-Flag monoclonal antibodies and goat anti-mouse IgG conjugated to 12-nm gold particles. Arrows point to the RSP2-Flag protein associated with the conjugative pilus. Bars represent 1 μm (A, B, and D) and 2 μm (C).

Taking into account the observed relationship between elements of the conjugation machinery of the R27 plasmid and the RSP and RSP2 proteins, we decided to assess whether these proteins, in addition to binding the flagella, they also target the conjugative pilus. To that end, we blocked flagellar expression and used electron microscopy in order to detect either the RSP or RSP2 protein bound to the conjugative pili. To prevent flagellar expression, we constructed Δ*fliC/fljB* (flagellin subunit) derivatives of strains SL1344(R27) and SL1344(R27 RSP2-Flag). Immunogold transmission electron microscopy imaging of these strains by using either polyclonal anti-RSP antibodies (47) or monoclonal anti-Flag antibodies showed that in both examples, gold particles were associated with tubular structures that likely corresponded to the conjugative pilus (Fig. 8A and C). The TrhH protein is required for pilus assembly, when the Δ*trhH* allele was introduced into the R27 plasmid of the mutant derivatives lacking flagella, generating the SL1344 Δ*fliC*Δ*fljB* (R27Δ*trhH*) and SL1344 Δ*fliC*Δ*fljB* (R27 RSP2-Flag Δ*trhH*) strains, the tubular structures were no longer detected, thus supporting the hypothesis that they corresponded to the conjugative pilus (Fig. 8B and D).

### The RSP2 protein is specific for IncHI1 plasmids.

The RSP protein is restricted to the IncHI plasmids, from both the IncHI1 and IncHI2 subgroups (47). Upon having shown the relevant role of the RSP2 protein in the conjugation of the IncHI1 plasmid R27, we performed a BLAST search to identify the *rsp2* gene in other bacterial plasmids (Table S2). In contrast to the RSP protein, the RSP2 protein is present in IncHI1 plasmids but not in IncHI2 plasmids. Interestingly, a group of IncN plasmids also contains a homolog of the R27 *rsp2* gene.

10.1128/msphere.00978-21.2 TABLE S2 Distribution of the RSP2 protein among bacterial plasmids. The results shown in the table correspond to a coverage greater than 99% and a percentage identity greater than 85%. The coverage of the rest of the results obtained fell to 36% or less. These results have not been considered. Download Table S2, XLSX file, 0.01 MB. Copyright © 2022 Hüttener et al. 2022 Hüttener et al. https://creativecommons.org/licenses/by/4.0/ This content is distributed under the terms of the Creative Commons Attribution 4.0 International license.

### Distribution of proteins containing the Big domain among bacterial plasmids.

We next addressed the question of whether proteins containing Big domains are a feature of only IncHI plasmids or whether they are also encoded by genes on plasmids of other incompatibility groups. We used for this analysis the genome viewer integrated into the NCBI database, configuring it to show the features and domains of the annotated proteins. The search was performed in assembled and sequenced plasmids of all incompatibility groups, selecting those that presented proteins with annotated Big domains. Among all the plasmids analyzed, proteins with these domains were also found in the plasmids of the IncA/C and IncP2 incompatibility groups. Big proteins from IncA/C plasmids show a high degree of similarity (see Fig. S1 in the supplemental material) and can be mapped to the corresponding *tra* regions (Fig. S2 and Fig. S3). Big proteins from IncP2 plasmids also show a very high degree of similarity (Fig. S4) and can be mapped close to a pilin gene (Fig. S3).

10.1128/msphere.00978-21.5 FIG S1 Alignment of proteins containing Big domains in IncA/C plasmids. Big 3_2 and Big3_3 domains are indicated by blue and green boxes, respectively. Download FIG S1, PDF file, 0.04 MB. Copyright © 2022 Hüttener et al. 2022 Hüttener et al. https://creativecommons.org/licenses/by/4.0/ This content is distributed under the terms of the Creative Commons Attribution 4.0 International license.

10.1128/msphere.00978-21.6 FIG S2 Genomic context of the identified Big proteins (in red) in IncA/C plasmids. The black boxes correspond to the Big 3.2 and Big 3.3 domains. Download FIG S2, PDF file, 0.1 MB. Copyright © 2022 Hüttener et al. 2022 Hüttener et al. https://creativecommons.org/licenses/by/4.0/ This content is distributed under the terms of the Creative Commons Attribution 4.0 International license.

10.1128/msphere.00978-21.7 FIG S3 Genomic context of the identified Big proteins (in red) in IncP2 plasmids. The black box corresponds to the Big 1 domain. Download FIG S3, PDF file, 0.1 MB. Copyright © 2022 Hüttener et al. 2022 Hüttener et al. https://creativecommons.org/licenses/by/4.0/ This content is distributed under the terms of the Creative Commons Attribution 4.0 International license.

10.1128/msphere.00978-21.8 FIG S4 Alignment of proteins containing the Big domain in IncP2 plasmids. The Big_1 domain is highlighted in orange. Download FIG S4, PDF file, 0.03 MB. Copyright © 2022 Hüttener et al. 2022 Hüttener et al. https://creativecommons.org/licenses/by/4.0/ This content is distributed under the terms of the Creative Commons Attribution 4.0 International license.

### The ALG87338.1 gene product of IncA/C plasmid pKAZ3 is required for plasmid conjugation at low temperature.

**FIG 9:**
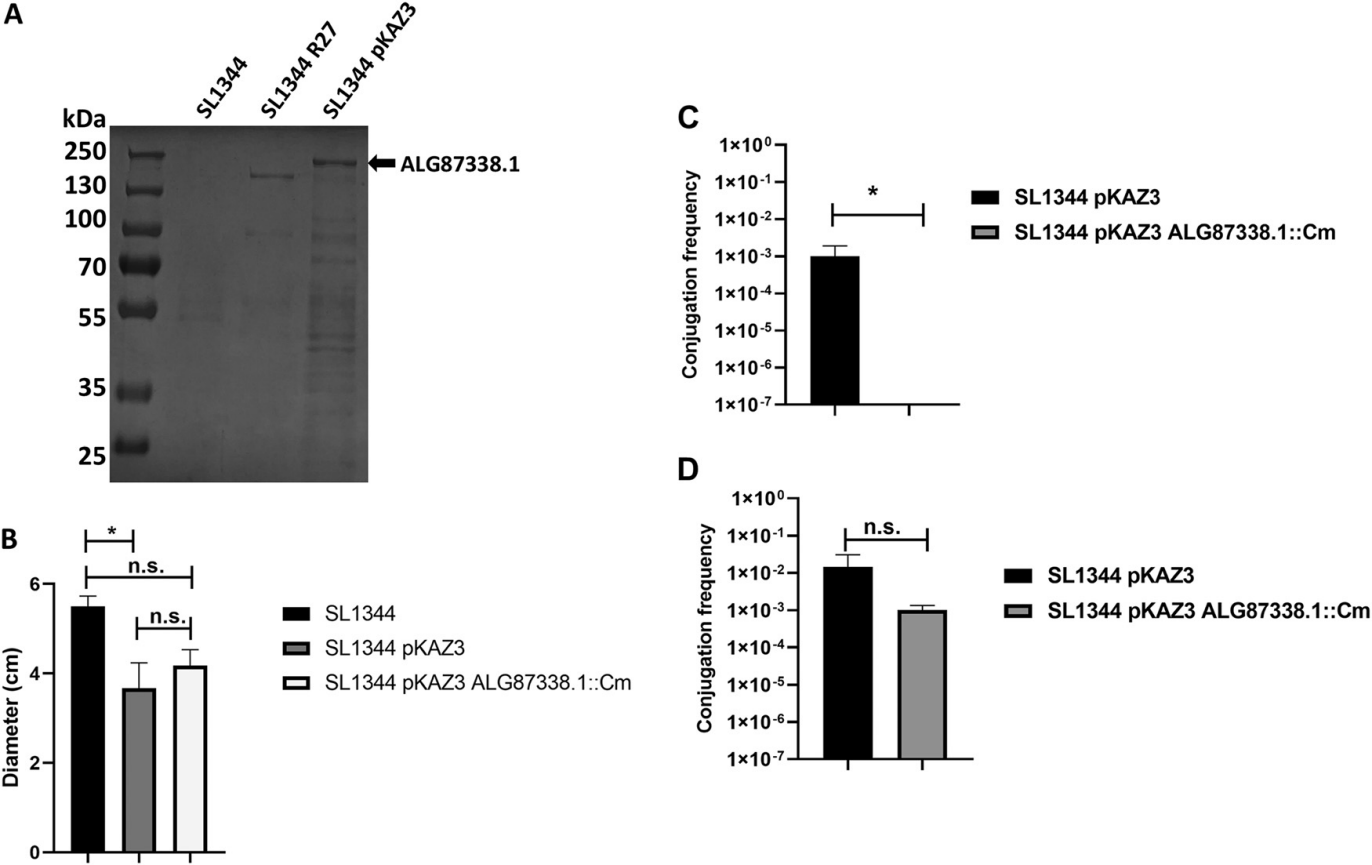
The ALG87388.1 gene product of the pKAZ3 plasmid influences conjugation at 25°C. (A) SDS-PAGE analysis of the cell-free secretomes of strains SL1344 wt, SL1344(R27), and SL1344(pKAZ3) grown at 25°C until the strain reached an OD_600_ of 2.0. The arrow points to the ALG87388.1 protein detected in the secretome of strain SL1344(pKAZ3) confirmed by LC-MS/MS. (B) Motility of the SL1344 wt, SL1344(pKAZ3), and SL1344(pKAZ3 ΔALG87388.1) strains. The motility of the different strains was measured as the halo diameter of the different colonies growing on motility agar. The data shown are the means plus standard deviations of three independent experiments. Statistical analysis showed significant differences analyzed by unpaired two-sided Student’s *t* test (*, *P* value = 0.0469; n.s., not significant). (C and D) Effect of the ΔALG87388.1 allele on the conjugation frequency of the pKAZ3 plasmid at 25°C (C) and 37°C (D) in liquid media. The data shown are the means plus standard deviations of three independent experiments. Statistical analysis showed significant differences analyzed by unpaired two-sided Student’s *t* test (*, *P* value = 0.042; n.s., not significant).

Upon having identified genes encoding proteins containing Big domains harbored on plasmids different from those of the IncHI group, we also aimed to assess whether a protein containing a Big domain encoded by a plasmid different from the IncHI group is also found in the secretome and influences conjugation. For this study, we selected IncA/C plasmid pKAZ3, carrying the ALG87338.1 gene, the product of which contains a Big domain. The pKAZ3 plasmid was isolated from an antibiotic-contaminated lake and confers multiple-antibiotic resistance (51). The ALG87338.1 gene product is an 1,843-aa protein (200.75 kDa) that contains Big (3_2 and 3_3) and DUF4165 domains (Fig. S5). Plasmid pKAZ3 was first transferred to strain SL1344, and the secretomes of strains SL1344 and SL1344(pKAZ3) were compared. A band corresponding to a protein of a molecular mass corresponding to the ALG87388.1 gene product was identified (Fig. 9A). This was confirmed by LC-MS/MS analysis of the protein excised from the SDS-PAGE. We next generated an ALG87388.1 knockout mutant derivative of plasmid pKAZ3. Considering that expression of either the RSP or RSP2 protein reduces SL1344 cell motility, we also compared the motility of strains SL1344, SL1344(pKAZ3), and SL1344(pKAZ3 ΔALG87388.1) (Fig. 9B). We observed that, while the acquisition of the pKAZ3 plasmid reduced cellular motility, there were no significant differences in motility between strains SL1344(pKAZ3) and SL1344(pKAZ3 ΔALG87388.1). We also compared the conjugation frequencies of strains SL1344(pKAZ3) and SL1344(pKAZ3 ΔALG87388.1) at both 37°C and at 25°C (Fig. 9C). ΔALG87388.1 did not influence the conjugation frequency at 37°C, but it had a very strong effect at 25°C.

10.1128/msphere.00978-21.9 FIG S5 Genomic context of the identified Big protein (ALG87338.1) in the IncA/C plasmid pKAZ3. The ALG87338.1 protein is an 1,843-aa protein and contains DUF4165, Big_3.2, and Big_3.3 domains. Download FIG S5, PDF file, 0.2 MB. Copyright © 2022 Hüttener et al. 2022 Hüttener et al. https://creativecommons.org/licenses/by/4.0/ This content is distributed under the terms of the Creative Commons Attribution 4.0 International license.

## DISCUSSION

The conjugative process has been studied for decades, and a detailed picture of the molecular mechanism of conjugational DNA transfer is available (as reviewed in references 50, 52, and 53). Plasmids such as the F factor, RP4, R388, or pTi have been studied as reference models to determine the function of several Tra proteins in plasmid conjugation by using genetic and biochemical approaches (50). Comprehensive information is available on processes such as conjugative pilus biosynthesis, the establishment of donor-recipient cell contact, or the assembly and activity of the relaxosome, which are key aspects of bacterial conjugation that must occur prior to the DNA transfer process. Nevertheless, several key questions, such as those regarding the function of several Tra proteins or the pilus’s ability to transport DNA between distant donor and recipient cells, remain to be answered (50). In this report, we have elaborated on the role of plasmid gene-encoded high-molecular-weight Big proteins in the conjugation process of different plasmids.

In the IncHI plasmid R27, two genes that were mapped to the Tra2 and Tra1-Tra2 intergenic regions (*rsp* and *rsp2*, respectively) (32, 33) encode proteins containing Big domains that participate in the conjugation process. Both the RSP and RSP2 proteins bind flagella, resulting in reduced cell motility. Export of the RSP2 protein to the external medium appears to be dependent upon RSP export. This may explain that, whereas loss of RSP function completely compensates for the effect of R27 acquisition on bacterial cell motility, loss of RSP2 function shows only a partial effect on cell motility.

The relationship between the RSP and RSP2 proteins and the conjugative machinery of the R27 plasmid is shown in this work. Expression of key elements required for the synthesis of the conjugative pilus of IncHI1 plasmids (TrhH protein) and of the pilin subunit itself (TrhA protein) is required for the correct translocation of the RSP and RSP2 proteins to the external surface of the cells. In addition, imaging of cells lacking flagella showed short filaments that likely corresponded to the conjugative pilus. These filaments were targeted both by the RSP and RSP2 proteins. Hence, there is an interaction between the RSP and RSP2 proteins and the R27 conjugative pilus.

IncHI plasmids and Salmonella participate in regulatory cross talk that is based on the expression of both the tetracycline determinant and other plasmid-borne genes (54, 55). One of the effects of the cross talk is the alteration in cellular motility, which is mediated by both plasmid-borne regulators (56) and the binding of the RSP and RSP2 proteins to the flagellar structure (47; this work). Nevertheless, these proteins also play a second and critical role in IncHI plasmid conjugation. They also bind the conjugative pilus, which is likely required to facilitate transmission of the conjugative plasmid. Plasmid-encoded Big proteins binding to the flagella and thus reducing motility may favor cell-to-cell contact and hence conjugation. Binding to the conjugative pilus may contribute to the stabilization of the pilus structure. The overall effect of expression of these proteins is to significantly favor the transfer of the plasmid that encodes them.

Genes encoding proteins containing Big domains are not restricted to IncHI plasmids. The genomic analysis performed in this work has shown that such proteins are also present in IncA/C and IncP2 plasmids. Both groups of plasmids have a wide host range. IncA/C plasmids are also key players in the dissemination of AMR in *Enterobacteriaceae* and other Gram-negative microorganisms. IncP2 plasmids are high-molecular-weight plasmids that are prevalent in Pseudomonas and contribute to the dissemination of AMR within this genus (57, 58). As the search that we performed had some limitations, it cannot be ruled out that other groups of plasmids also express Big proteins. By using the genome viewer integrated into the NCBI database, only those plasmids that were well annotated and characterized were considered. In addition, proteins that contain Big domains were annotated because of the confirmation that this domain was present, not because they shared amino acid sequence similarity. Notably, the RSP2 protein is specific of a subgroup of IncHI plasmids, IncHI1. IncHI2 plasmids that carry the *rsp* gene lack the *rsp2* gene. Interestingly, our BLAST analysis also showed that the *rsp2* gene has jumped to plasmids of a different Inc group, IncN. This may indicate that the *rsp2* genes are spreading and may also influence conjugation in these plasmids.

The identified high-molecular-weight Big proteins encoded by genes on IncA/C plasmids show a very high degree of homology, which suggests that they play similar roles in these plasmids. The genes encoding these proteins were also mapped to the *tra* region (*traV*/*A*/*W*/*F*/*N* genes) and, in some instances, were adjacent to the pilin genes. A recent study focused on performing a comprehensive analysis of IncC plasmid conjugation (59), and the gene encoding the identified Big protein carried on the pMS6198A plasmid (the product of the gene MS6198_A094) was considered not to influence conjugation, despite mapping within the Tra1 region, between the *dsbCA* and *traL* genes. The likely reason for this was that the conjugation experiments were performed only at 37°C. We showed here that the ALG87338.1 gene product of the IncA/C plasmid pKAZ3 positively influenced conjugation at 25°C (i.e., at environmental temperature). IncHI and IncA/C plasmids belong to the same relaxase family (MOB_H_). Interestingly, they also share the requirement for a high-molecular-weight Big protein for efficient conjugation at environmental temperatures. The identified Big proteins of IncP2 plasmids also show a high degree of similarity. While the molecular masses of these proteins are lower than those of the Big proteins of IncHI and IncA/C plasmids, they map close to a pilin, which also suggests that they may play a role in the conjugative process.

Out of the IncHI plasmids, which have classically been characterized as being conjugative only at temperatures below 30°C, plasmids harbored by *Enterobacteriaceae* have usually been studied in cells growing at the optimal growth temperature for these microorganisms, that is, 37°C. Our study also highlights the importance of studying plasmid conjugation at other temperatures, closer to those that most microorganisms encounter outside their warm-blooded hosts. Indeed, AMR transmission occurs mainly in natural environments at temperatures that rarely reach 37°C.

The relevance of the Big proteins studied in this work is not only their role as elements favoring plasmid conjugation but also the fact that, as they are associated with extracellular appendages of the bacterial cells, they can be targeted with specific antibodies either to restrict the dissemination of the plasmids that encode the genes or, when expressed within the human body, to control infections caused by bacteria that express the plasmid that carries the genes that encode these proteins.

## MATERIALS AND METHODS

### Bacterial strains, plasmids, and growth conditions.

The bacterial strains (see Table S3 in the supplemental material) were routinely grown in Luria-Bertani (LB) medium (10 g L^−1^ NaCl, 10 g L^−1^ tryptone, and 5 g L^−1^ yeast extract) with vigorous shaking at 200 rpm (Innova 3100; New Brunswick Scientific). The antibiotics used were chloramphenicol (Cm) (25 μg mL^−1^), tetracycline (Tc) (15 μg mL^−1^), carbenicillin (Cb) (100 μg mL^−1^), and kanamycin (Km) (50 μg mL^−1^) (Sigma-Aldrich).

10.1128/msphere.00978-21.3 TABLE S3 Bacterial strains and plasmids used in this work. Download Table S3, DOCX file, 0.04 MB. Copyright © 2022 Hüttener et al. 2022 Hüttener et al. https://creativecommons.org/licenses/by/4.0/ This content is distributed under the terms of the Creative Commons Attribution 4.0 International license.

### Oligonucleotides.

The oligonucleotides (from 5′ to 3′) used in this work are listed in Table S4.

10.1128/msphere.00978-21.4 TABLE S4 Oligonucleotides used in this study. Download Table S4, DOCX file, 0.01 MB. Copyright © 2022 Hüttener et al. 2022 Hüttener et al. https://creativecommons.org/licenses/by/4.0/ This content is distributed under the terms of the Creative Commons Attribution 4.0 International license.

### Genetic manipulation.

All enzymes used to perform standard molecular and genetic procedures were used according to the manufacturer’s recommendations. To introduce plasmids into E. coli and Salmonella, bacterial cells were grown until an OD_600_ of 0.6. Cells were then washed several times with 10% glycerol, and the respective plasmids or DNA was electroporated by using an Eppendorf gene pulser (Electroporator 2510).

Deletions of the *rsp2* (ORF *R0055*), *fliC*, *fljB*, *trhH*, and *trhA* genes were performed in strain SL1344(R27) by using the λ Red recombination method as previously described (60). The antibiotic resistance determinant of the plasmids pKD3/pKD4 was amplified using the corresponding oligonucleotides (P1/P2 series [Table S4]). The mutants were confirmed by PCR using the corresponding oligonucleotides (P1up/P2down series [Table S4]). We used phage P22 HT for combining mutations by transduction (61). When necessary, the antibiotic resistance cassette was eliminated using the FLP/FRT recombination target (FRT)-mediated site-specific recombination method as previously described (62).

For deletion of the ALG87338.1 gene from the pKAZ3 plasmid, we first constructed the plasmid pKD46-Km^R^ following the strategy described in reference 63. Briefly, kanamycin resistance from the pKD4 plasmid was amplified using the PkmXmnIFW/PkmXmnIRv oligonucleotides together with Phusion Hot Start II High-Fidelity DNA polymerase (Thermo Scientific) following the manufacturer’s recommendations. The corresponding XmnI-flanked kanamycin resistance fragment was cloned into the vector pKD46 previously digested with the same enzyme. The resulting plasmid was termed pKD46-Km^R^. Deletion of ALG87338.1 was performed in strain SL1344(pKAZ3) pKD46-Km^R^ by using the λ Red recombination method. The antibiotic resistance determinant of the pKD3 plasmid was amplified using the corresponding oligonucleotides (P1/P2 series [Table S4]). The mutants were confirmed by PCR using the corresponding oligonucleotides (P1up/P2down series [Table S4]).

Recombinational transfer of the Flag sequence into the *rsp2* gene was achieved by following a previously described methodology (64). The template vector encoding Flag and Km^r^ used was the pSUB11 plasmid. The primers used for construction of the Flag-tagged derivative were R27_p0553XP1 and R27_p0553XP2 (Table S4). The correct insertion of the Flag tag was confirmed by PCR using oligonucleotides R27_p0553XP1UP and R27_p0553XP2DOWN (Table S4).

To construct the pLG338-*rsp2* plasmid, the ORF *R0055* (*rsp2*) (GenBank accession number AF250878.1, positions 56639 to 59970) was amplified using the oligonucleotides R27_R55 pLG322 ECORI fw/R27_R55 pLG322 Bam rv (see Table S4 for the sequences) together with Phusion Hot Start II High-Fidelity DNA polymerase (Thermo Scientific) following the manufacturer’s recommendations. The corresponding EcoRI/BamHI fragment was cloned into the pLG338-30 vector previously digested with the same enzymes. The resulting plasmids were Sanger sequenced and termed pLG338-*rsp2*.

### Plasmid conjugation.

The R27 and pKAZ3 plasmids were conjugated as described previously (47). The mating frequency was calculated as the number of transconjugants per donor cell. Student’s *t* test was used to determine statistical significance, and the values were obtained by using GraphPad Prism 8 software. A *P* value of less than 0.05 was considered significant.

### Motility assays.

The motility assay was performed as previously described (47). The experiments were repeated three times with three plates of each strain in each experiment. The colony diameter was measured and plotted, and standard errors were calculated. Student’s *t* test was used to determine statistical significance, and the values were obtained by using the GraphPad Prism 8 software. A *P* value of less than 0.05 was considered significant.

### Flagellum isolation.

Flagellum isolation was prepared as previously described (47).

### Cell-free secreted proteins (secretome).

Cell-free supernatants were prepared as previously described (47).

### Cell fractionation.

Cell fractionation was performed as previously described (47).

### Immunogold electron microscopy.

Immunogold microscopy experiments were performed as previously described (47).

### Electrophoresis and Western blotting of proteins.

Protein samples were analyzed by 10% and 12.5% SDS-PAGE (65). Proteins were transferred from the gels to polyvinylidene difluoride (PVDF) membranes using the Trans-Blot Turbo system (Bio-Rad). Western blot analysis was performed with a monoclonal antibody raised against the Flag epitope (Sigma) diluted 1:10,000 in a solution containing phosphate-buffered saline (PBS), 0.2% Triton, and 3% skim milk and incubated for 16 h at 4°C. The membranes were washed for 20 min each with PBS and 0.2% Triton solution. The washing step was repeated three times. Thereafter, the membranes were incubated with horseradish peroxidase-conjugated goat anti-mouse IgG (Invitrogen) diluted 1:5,000 in a solution containing PBS and 0.2% Triton for 1 h at room temperature. Again, three washing steps of 45 min with PBS and 0.2% Triton solution were performed, and detection was performed by enhanced chemiluminescence using ImageQuant LAS 54000 imaging system software (GE Healthcare Lifesciences).

### RSP-Flag immunoprecipitation.

For RSP-Flag protein immunoprecipitation, strains SL1344(R27 RSP-Flag) and SL1344(R27 Δ*rsp*, negative control) were grown in LB medium for 16 h at 37°C. One hundred milliliters of fresh LB medium was inoculated 1:100 with both overnight-cultured strains and grown at 25°C until an OD_600_ of 2.0 was reached. Then, the cells were centrifuged at 9,000 rpm for 30 min at 4°C, the pellets were discarded, and the supernatants were filtered through 0.22-μm filters. For each immunoprecipitation protocol, we used 100 μl of anti-Flag M2 affinity gel (Sigma-Aldrich) and 100 ml of each supernatant and incubated the mixture under slow rotation at 4°C for 16 h. Each supernatant was loaded onto a Poly-Prep chromatography column (Bio-Rad), and the flowthrough was stored at −20°C for further analysis. Each column was washed out with 100 ml of washing buffer (50 mM Tris-HCl [pH 7.5], 150 mM NaCl), and elution was performed three times with 0.3 ml of elution buffer (0.1 M glycine [pH 3.5]). Elution fractions were concentrated using a trichloroacetic acid (TCA) precipitation protocol. Briefly, 1 ml of the samples was mixed with 0.5 ml of a 45% TCA solution (wt/vol). The samples were kept on ice for 30 min and centrifuged for 30 min at 13,400 rpm. The supernatants were carefully discarded, acetone was added to the protein pellets, and the pellets were again centrifuged for 30 min at 13,400 rpm. The supernatants were carefully discarded. The protein pellets were dried and solubilized with 1× Laemmli sample buffer (Bio-Rad). Samples were boiled for 10 min and loaded onto a 12.5% SDS-PAG. When the protein samples entered the stacking phase, the gel run was stopped, and samples were excised from the gel and sent to the Proteomic Platform (Barcelona Science Park, Barcelona, Spain) for protein identification (55). Immunoprecipitation and protein identification experiments were repeated twice.

## References

[1] Morens DM, Folkers GK, Fauci AS. 2004. The challenge of emerging and re-emerging infectious diseases. Nature 430:242–249. doi:10.1038/nature02759. 15241422 PMC7094993

[2] Meyer E, Schwab F, Schroeren-Boersch B, Gastmeier P. 2010. Dramatic increase of third-generation cephalosporin-resistant E. coli in German intensive care units: secular trends in antibiotic drug use and bacterial resistance, 2001 to 2008. Crit Care 14:R113. doi:10.1186/cc9062. 20546564 PMC2911759

[3] Rossolini GM, Mantengoli E, Docquier J-D, Musmanno RA, Coratza G. 2007. Epidemiology of infections caused by multiresistant Gram-negatives: ESBLs, MBLs, panresistant strains. New Microbiol 30:332–339. 17802921

[4] Spellberg B, Guidos R, Gilbert D, Bradley J, Boucher HW, Scheld WM, Bartlett JG, Edwards J, Infectious Diseases Society of America. 2008. The epidemic of antibiotic-resistant infections: a call to action for the medical community from the Infectious Diseases Society of America. Clin Infect Dis 46:155–164. doi:10.1086/524891. 18171244

[5] Carattoli A. 2013. Plasmids and the spread of resistance. Int J Med Microbiol 303:298–304. doi:10.1016/j.ijmm.2013.02.001. 23499304

[6] Wang J, Stephan R, Zurfluh K, Hächler H, Fanning S. 2015. Characterization of the genetic environment of *bla*_ESBL_ genes, integrons and toxin-antitoxin systems identified on large transferrable plasmids in multi-drug resistant *Escherichia coli*. Front Microbiol 6:716. doi:10.3389/fmicb.2014.00716. 25610429 PMC4285173

[7] Thomas CM, Nielsen KM. 2005. Mechanisms of, and barriers to, horizontal gene transfer between bacteria. Nat Rev Microbiol 3:711–721. doi:10.1038/nrmicro1234. 16138099

[8] Vrancianu CO, Popa LI, Bleotu C, Chifiriuc MC. 2020. Targeting plasmids to limit acquisition and transmission of antimicrobial resistance. Front Microbiol 11:761. doi:10.3389/fmicb.2020.00761. 32435238 PMC7219019

[9] Carattoli A. 2009. Resistance plasmid families in Enterobacteriaceae. Antimicrob Agents Chemother 53:2227–2238. doi:10.1128/AAC.01707-08. 19307361 PMC2687249

[10] Novick RP. 1987. Plasmid incompatibility. Microbiol Rev 51:381–395. doi:10.1128/mr.51.4.381-395.1987. 3325793 PMC373122

[11] Garcillán-Barcia MP, Francia MV, de La Cruz F. 2009. The diversity of conjugative relaxases and its application in plasmid classification. FEMS Microbiol Rev 33:657–687. doi:10.1111/j.1574-6976.2009.00168.x. 19396961

[12] Phan MD, Wain J. 2008. IncHI plasmids, a dynamic link between resistance and pathogenicity. J Infect Dev Ctries 30:272–278. doi:10.3855/jidc.221. 19741288

[13] Gilmour MW, Thomson NR, Sanders M, Parkhill J, Taylor DE. 2004. The complete nucleotide sequence of the resistance plasmid R478: defining the backbone components of incompatibility group H conjugative plasmids through comparative genomics. Plasmid 52:182–202. doi:10.1016/j.plasmid.2004.06.006. 15518875

[14] Forde BM, Zowawi HM, Harris PNA, Roberts L, Ibrahim E, Shaikh N, Deshmukh A, Sid Ahmed MA, Al Maslamani M, Cottrell K, Trembizki E, Sundac L, Yu HH, Li J, Schembri MA, Whiley DM, Paterson DL, Beatson SA. 2018. Discovery of mcr-1 -mediated colistin resistance in a highly virulent *Escherichia coli* lineage. mSphere 3:e00486-18. doi:10.1128/mSphere.00486-18. 30305321 PMC6180223

[15] Villa L, Poirel L, Nordmann P, Carta C, Carattoli A. 2012. Complete sequencing of an IncH plasmid carrying the *bla*_ndm-1_, *bla*_ctx-m-15_ and *qnrB1* genes. J Antimicrob Chemother 67:1645–1650. doi:10.1093/jac/dks114. 22511638

[16] Dolejska M, Villa L, Poirel L, Nordmann P, Carattoli A. 2013. Complete sequencing of an IncHI1 plasmid encoding the carbapenemase NDM-1, the ArmA 16S RNA methylase and a resistance-nodulation-cell division/multidrug efflux pump. J Antimicrob Chemother 68:34–39. doi:10.1093/jac/dks357. 22969080

[17] Holt KE, Phan MD, Baker S, Duy PT, Nga TVT, Nair S, Turner AK, Walsh C, Fanning S, Farrell-Ward S, Dutta S, Kariuki S, Weill F-X, Parkhill J, Dougan G, Wain J. 2011. Emergence of a globally dominant IncHI1 plasmid type associated with multiple drug resistant typhoid. PLoS Negl Trop Dis 5:e1245. doi:10.1371/journal.pntd.0001245. 21811646 PMC3139670

[18] Lim LM, Ly N, Anderson D, Yang JC, Macander L, Jarkowski A, Forrest A, Bulitta JB, Tsuji BT. 2010. Resurgence of colistin: a review of resistance, toxicity, pharmacodynamics, and dosing. Pharmacotherapy 30:1279–1291. doi:10.1592/phco.30.12.1279. 21114395 PMC4410713

[19] Catry B, Cavaleri M, Baptiste K, Grave K, Grein K, Holm A, Jukes H, Liebana E, Navas AL, Mackay D, Magiorakos AP, Romo MAM, Moulin G, Madero CM, Pomba MCMF, Powell M, Pyörälä S, Rantala M, Ružauskas M, Sanders P, Teale C, Threlfall EJ, Törneke K, Van Duijkeren E, Edo JT. 2015. Use of colistin-containing products within the European Union and European Economic Area (EU/EEA): development of resistance in animals and possible impact on human and animal health. Int J Antimicrob Agents 46:297–306. doi:10.1016/j.ijantimicag.2015.06.005. 26215780

[20] Olaitan AO, Morand S, Rolain JM. 2014. Mechanisms of polymyxin resistance: acquired and intrinsic resistance in bacteria. Front Microbiol 5:643. doi:10.3389/fmicb.2014.00643. 25505462 PMC4244539

[21] Liu Y-Y, Wang Y, Walsh TR, Yi L-X, Zhang R, Spencer J, Doi Y, Tian G, Dong B, Huang X, Yu L-F, Gu D, Ren H, Chen X, Lv L, He D, Zhou H, Liang Z, Liu J-H, Shen J. 2016. Emergence of plasmid-mediated colistin resistance mechanism MCR-1 in animals and human beings in China: a microbiological and molecular biological study. Lancet Infect Dis 16:161–168. doi:10.1016/S1473-3099(15)00424-7. 26603172

[22] Gao R, Hu Y, Li Z, Sun J, Wang Q, Lin J, Ye H, Liu F, Srinivas S, Li D, Zhu B, Liu YH, Tian GB, Feng Y. 2016. Dissemination and mechanism for the MCR-1 colistin resistance. PLoS Pathog 12:e1005957. doi:10.1371/journal.ppat.1005957. 27893854 PMC5125707

[23] Xavier BB, Lammens C, Ruhal R, Malhotra-Kumar S, Butaye P, Goossens H, Malhotra-Kumar S. 2016. Identification of a novel plasmid-mediated colistin-resistance gene, *mcr-2*, in *Escherichia coli*, Belgium, June 2016. Euro Surveill 21(27):pii=30280. 10.2807/1560-7917.ES.2016.21.27.30280. 27416987

[24] Borowiak M, Fischer J, Hammerl JA, Hendriksen RS, Szabo I, Malorny B. 2017. Identification of a novel transposon-associated phosphoethanolamine transferase gene, *mcr-5*, conferring colistin resistance in d-tartrate fermenting *Salmonella enterica* subsp. enterica serovar Paratyphi B. J Antimicrob Chemother 72:3317–3324. doi:10.1093/jac/dkx327. 28962028

[25] Carattoli A, Villa L, Feudi C, Curcio L, Orsini S, Luppi A, Pezzotti G, Magistrali CF. 2017. Novel plasmid-mediated colistin resistance *mcr-4* gene in *Salmonella* and *Escherichia coli*, Italy 2013, Spain and Belgium, 2015 to 2016. Euro Surveill 22(31):pii=30589. 10.2807/1560-7917.ES.2017.22.31.30589. PMC5553062 28797329

[26] Yin W, Li H, Shen Y, Liu Z, Wang S, Shen Z, Zhang R, Walsh TR, Shen J, Wang Y. 2021. Novel plasmid-mediated colistin resistance gene mcr-3 in *Escherichia coli*. mBio 8:e00543-17. doi:10.1128/mBio.00543-17. PMC5487729 28655818

[27] Matamoros S, Van Hattem JM, Arcilla MS, Willemse N, Melles DC, Penders J, Vinh TN, Thi Hoa N, Bootsma MCJ, Van Genderen PJ, Goorhuis A, Grobusch M, Molhoek N, Oude Lashof AML, Stobberingh EE, Verbrugh HA, De Jong MD, Schultsz C. 2017. Global phylogenetic analysis of *Escherichia coli* and plasmids carrying the *mcr-1* gene indicates bacterial diversity but plasmid restriction. Sci Rep 7:15364. doi:10.1038/s41598-017-15539-7. 29127343 PMC5681592

[28] Wang Y, Tian G-B, Zhang R, Shen Y, Tyrrell JM, Huang X, Zhou H, Lei L, Li H-Y, Doi Y, Fang Y, Ren H, Zhong L-L, Shen Z, Zeng K-J, Wang S, Liu J-H, Wu C, Walsh TR, Shen J. 2017. Prevalence, risk factors, outcomes, and molecular epidemiology of mcr-1-positive Enterobacteriaceae in patients and healthy adults from China: an epidemiological and clinical study. Lancet Infect Dis 17:390–399. doi:10.1016/S1473-3099(16)30527-8. 28139431

[29] Zheng B, Dong H, Xu H, Lv J, Zhang J, Jiang X, Du Y, Xiao Y, Li L. 2016. Coexistence of MCR-1 and NDM-1 in clinical *Escherichia coli* isolates. Clin Infect Dis 63:1393–1395. doi:10.1093/cid/ciw553. 27506685

[30] Maher D, Sherburne R, Taylor DE. 1993. H-pilus assembly kinetics determined by electron microscopy. J Bacteriol 175:2175–2183. doi:10.1128/jb.175.8.2175-2183.1993. 8096837 PMC204501

[31] Alonso G, Baptista K, Ngo T, Taylor DE. 2005. Transcriptional organization of the temperature-sensitive transfer system from the IncHI1 plasmid R27. Microbiology (Reading) 151:3563–3573. doi:10.1099/mic.0.28256-0. 16272379

[32] Lawley TD, Gilmour MW, Gunton JE, Standeven LJ, Taylor DE. 2002. Functional and mutational analysis of conjugative transfer region 1 (Tra1) from the IncHI1 plasmid R27. J Bacteriol 184:2173–2180. doi:10.1128/JB.184.8.2173-2180.2002. 11914349 PMC134963

[33] Lawley TD, Gilmour MW, Gunton JE, Tracz DM, Taylor DE. 2003. Functional and mutational analysis of conjugative transfer region 2 (Tra2) from the IncHI1 plasmid R27. J Bacteriol 185:581–591. doi:10.1128/JB.185.2.581-591.2003. 12511505 PMC145343

[34] Sherburne CK, Lawley TD, Gilmour MW, Blattner FR, Burland V, Grotbeck E, Rose DJ, Taylor DE. 2000. The complete DNA sequence and analysis of R27, a large IncHI plasmid from *Salmonella* typhi that is temperature sensitive for transfer. Nucleic Acids Res 28:2177–2186. doi:10.1093/nar/28.10.2177. 10773089 PMC105367

[35] Aoki T, Egusa S, Kimura T, Watanabe T. 1971. Detection of R factors in naturally occurring *Aeromonas salmonicida* strains. Appl Microbiol 22:716–717. doi:10.1128/am.22.4.716-717.1971. 4108649 PMC376391

[36] Watanabe T, Aoki T, Ogata Y, Egusa S. 1971. R factors related to fish culturing. Ann N Y Acad Sci 182:383–410. doi:10.1111/j.1749-6632.1971.tb30674.x. 4936671

[37] Welch TJ, Fricke WF, McDermott PF, White DG, Rosso ML, Rasko DA, Mammel MK, Eppinger M, Rosovitz MJ, Wagner D, Rahalison L, LeClerc JE, Hinshaw JM, Lindler LE, Cebula TA, Carniel E, Ravel J. 2007. Multiple antimicrobial resistance in plague: an emerging public health risk. PLoS One 2:e309. doi:10.1371/journal.pone.0000309. 17375195 PMC1819562

[38] Fricke WF, Welch TJ, McDermott PF, Mammel MK, LeClerc JE, White DG, Cebula TA, Ravel J. 2009. Comparative genomics of the IncA/C multidrug resistance plasmid family. J Bacteriol 191:4750–4757. doi:10.1128/JB.00189-09. 19482926 PMC2715731

[39] Call DR, Singer RS, Meng D, Broschat SL, Orfe LH, Anderson JM, Herndon DR, Kappmeyer LS, Daniels JB, Besser TE. 2010. blaCMY-2-positive IncA/C plasmids from *Escherichia coli* and *Salmonella enterica* are a distinct component of a larger lineage of plasmids. Antimicrob Agents Chemother 54:590–596. doi:10.1128/AAC.00055-09. 19949054 PMC2812137

[40] Fernández-Alarcón C, Singer RS, Johnson TJ. 2011. Comparative genomics of multidrug resistance-encoding IncA/C plasmids from commensal and pathogenic *Escherichia coli* from multiple animal sources. PLoS One 6:e23415. doi:10.1371/journal.pone.0023415. 21858108 PMC3155540

[41] Suzuki H, Yano H, Brown CJ, Top EM. 2010. Predicting plasmid promiscuity based on genomic signature. J Bacteriol 192:6045–6055. doi:10.1128/JB.00277-10. 20851899 PMC2976448

[42] Eda R, Nakamura M, Takayama Y, Maehana S, Nakano R, Yano H, Kitasato H. 2020. Trends and molecular characteristics of carbapenemase-producing Enterobacteriaceae in Japanese hospital from 2006 to 2015. J Infect Chemother 26:667–671. doi:10.1016/j.jiac.2020.02.002. 32222331

[43] Rozwandowicz M, Brouwer MSM, Fischer J, Wagenaar JA, Gonzalez-Zorn B, Guerra B, Mevius DJ, Hordijk J. 2018. Plasmids carrying antimicrobial resistance genes in Enterobacteriaceae. J Antimicrob Chemother 73:1121–1137. doi:10.1093/jac/dkx488. 29370371

[44] Qamar MU, Ejaz H, Walsh TR, Shah AA, Al Farraj DA, Alkufeidy RM, Alkubaisi NA, Saleem S, Jahan S. 2021. Clonal relatedness and plasmid profiling of extensively drug-resistant New Delhi metallo-β-lactamase-producing *Klebsiella pneumoniae* clinical isolates. Future Microbiol 16:229–239. doi:10.2217/fmb-2020-0315. 33625250

[45] Halaby DM, Mornon JPE. 1998. The immunoglobulin superfamily: an insight on its tissular, species, and functional diversity. J Mol Evol 46:389–400. doi:10.1007/pl00006318. 9541533

[46] Bodelón G, Palomino C, Fernández LÁ. 2013. Immunoglobulin domains in *Escherichia coli* and other enterobacteria: from pathogenesis to applications in antibody technologies. FEMS Microbiol Rev 37:204–250. doi:10.1111/j.1574-6976.2012.00347.x. 22724448

[47] Hüttener M, Prieto A, Aznar S, Bernabeu M, Glaría E, Valledor AF, Paytubi S, Merino S, Tomás J, Juárez A. 2019. Expression of a novel class of bacterial Ig-like proteins is required for IncHI plasmid conjugation. PLoS Genet 15:e1008399. doi:10.1371/journal.pgen.1008399. 31527905 PMC6764697

[48] Arutyunov D, Arenson B, Manchak J, Frost LS. 2010. F plasmid TraF and TraH are components of an outer membrane complex involved in conjugation. J Bacteriol 192:1730–1734. doi:10.1128/JB.00726-09. 20081027 PMC2832511

[49] Frost LS, Ippen-Ihler K, Skurray RA. 1994. Analysis of the sequence and gene products of the transfer region of the F sex factor. Microbiol Mol Biol Rev 58:162–210. 10.1128/mr.58.2.162-210.1994 PMC372961 7915817

[50] Virolle C, Goldlust K, Djermoun S, Bigot S, Lesterlin C. 2020. Plasmid transfer by conjugation in Gram-negative bacteria: from the cellular to the community level. Genes 11:1239. doi:10.3390/genes11111239. PMC7690428 33105635

[51] Flach CF, Johnning A, Nilsson I, Smalla K, Kristiansson E, Larsson DGJ. 2015. Isolation of novel IncA/C and IncN fluoroquinolone resistance plasmids from an antibiotic-polluted lake. J Antimicrob Chemother 70:2709–2717. doi:10.1093/jac/dkv167. 26124213

[52] Smillie C, Garcillán-Barcia MP, Francia MV, Rocha EPC, de la Cruz F. 2010. Mobility of plasmids. Microbiol Mol Biol Rev 74:434–452. doi:10.1128/MMBR.00020-10. 20805406 PMC2937521

[53] Cabezón E, Ripoll-Rozada J, Peña A, de la Cruz F, Arechaga I. 2015. Towards an integrated model of bacterial conjugation. FEMS Microbiol Rev 39:81–95. doi:10.1111/1574-6976.12085. 25154632

[54] Paytubi S, Aznar S, Madrid C, Balsalobre C, Dillon SC, Dorman CJ, Juárez A. 2014. A novel role for antibiotic resistance plasmids in facilitating *Salmonella* adaptation to non-host environments. Environ Microbiol 16:950–962. doi:10.1111/1462-2920.12244. 24024872

[55] Hüttener M, Prieto A, Aznar S, Dietrich M, Paytubi S, Juárez A. 2018. Tetracycline alters gene expression in *Salmonella* strains that harbor the Tn10 transposon. Environ Microbiol Rep 10:202–209. doi:10.1111/1758-2229.12621. 29393572

[56] Luque A, Paytubi S, Sánchez-Montejo J, Gibert M, Balsalobre C, Madrid C. 2019. Crosstalk between bacterial conjugation and motility is mediated by plasmid-borne regulators. Environ Microbiol Rep 11:708–717. doi:10.1111/1758-2229.12784. 31309702

[57] Jacoby GA, Sutton L, Knobel L, Mammen P. 1983. Properties of IncP-2 plasmids of *Pseudomonas* spp. Antimicrob Agents Chemother 24:168–175. doi:10.1128/AAC.24.2.168. 6638986 PMC185132

[58] Cazares A, Moore MP, Hall JPJ, Wright LL, Grimes M, Emond-Rhéault JG, Pongchaikul P, Santanirand P, Levesque RC, Fothergill JL, Winstanley C. 2020. A megaplasmid family driving dissemination of multidrug resistance in *Pseudomonas*. Nat Commun 11:1370. doi:10.1038/s41467-020-15081-7. 32170080 PMC7070040

[59] Hancock SJ, Phan MD, Luo Z, Lo AW, Peters KM, Nhu NTK, Forde BM, Whitfield J, Yang J, Strugnell RA, Paterson DL, Walsh TR, Kobe B, Beatson SA, Schembri MA. 2020. Comprehensive analysis of IncC plasmid conjugation identifies a crucial role for the transcriptional regulator AcaB. Nat Microbiol 5:1340–1348. doi:10.1038/s41564-020-0775-0. 32807890

[60] Datsenko KA, Wanner BL. 2000. One-step inactivation of chromosomal genes in *Escherichia coli* K-12 using PCR products. Proc Natl Acad Sci USA 97:6640–6645. doi:10.1073/pnas.120163297. 10829079 PMC18686

[61] Sternberg NL, Maurer R. 1991. Bacteriophage-mediated generalized transduction in *Escherichia coli* and *Salmonella* Typhimurium. Methods Enzymol 204:18–43. doi:10.1016/0076-6879(91)04004-8. 1943777

[62] Cherepanov PP, Wackernagel W. 1995. Gene disruption in *Escherichia coli*: TcR and KmR cassettes with the option of Flp-catalyzed excision of the antibiotic-resistance determinant. Gene 158:9–14. doi:10.1016/0378-1119(95)00193-a. 7789817

[63] Doublet B, Douard G, Targant H, Meunier D, Madec JY, Cloeckaert A. 2008. Antibiotic marker modifications of λ Red and FLP helper plasmids, pKD46 and pCP20, for inactivation of chromosomal genes using PCR products in multidrug-resistant strains. J Microbiol Methods 75:359–361. doi:10.1016/j.mimet.2008.06.010. 18619499

[64] Uzzau S, Figueroa-Bossi N, Rubino S, Bossi L. 2001. Epitope tagging of chromosomal genes in *Salmonella*. Proc Natl Acad Sci USA 98:15264–15269. doi:10.1073/pnas.261348198. 11742086 PMC65018

[65] Sambrook J, Russell D. 2001. Molecular cloning: a laboratory manual, 3rd ed. Cold Spring Harbor Laboratory Press, Cold Spring Harbor, NY.

