# Supplementary material for: Roles of Proteins Containing Immunoglobulin-Like Domains in the Conjugation of Bacterial Plasmids
Source: mSphere. 2022 Jan 5;7(1):e00978-21. doi: 10.1128/msphere.00978-21 (PMC8730810; doi:10.1128/msphere.00978-21)
Supplement: FIG S1 [file msphere.00978-21-sf001.pdf]

```
pKAZ3           ----MINFKPKIPAMLGALAVLTAGAAHAELLEYTFKAPDGTQRSLTPNANYANPTGNIS 56
pMS6198A        ----MINFKPKIPAMLGALAVLTAGAAHAELLEYTFKAPDGTQRSLTPNANYANPTGNIS 56
pKp55           MENAMINFKPKIPAMLGALAVLTAGAAHAELLEYTFKAPDGTQRSLTPNANYANPTGNIS 60
pEc19           MENAMINFKPKIPAMLGALAVLTAGAAHAELLEYTFKAPDGTQRSLTPNANYANPTGNIS 60
pAPEC1990       ----MINFKPKIPAMLGALAVLTAGAAHAELLEYTFKAPDGTQRSLTPNANYANPTGNIS 56
pNDM-KN         ----MINFKPKIPAMLGALAVLTAGAAHAELLEYTFKAPDGTQRSLTPNANYANPTGNIS 56
pUMNK88         ----MINFKPKIPAMLGALAVLTAGAAHAELLEYTFKAPDGTQRSLTPNANYANPTGNIS 56
pCf587          ----MINFKPKLPALLGALAVLTAGSAHAELLEYTFKAPDGAQRSLPPNANYANPTGNVS 56
                    *******:**:**********:***************:**** ***********:*

pKAZ3           FALSAGIDRKVKISVIRSDGTVVSTATSHLLGATDRITVGGKSYYGAELQLPAPSEGVYK 116
pMS6198A        FALSAGIDRKVKISVLRSDGTVVSTATSHLLGATDRITVGGKSYYGAELQLPAPVGGAYT 116
pKp55           FALSAGIDRKVKISVLRSDGTVVSTATSHLLGATDRITVGGKSYYGAELQLPAPVGGAYT 120
pEc19           FALSAGIDRKVKISVLRSDGTVVSTATSHLLGATDRITVGGKSYYGAELQLPAPVGGAYT 120
pAPEC1990       FALSAGIDRKVKISVLRSDGTVVSTATSHLLGATDRITVGGKSYYGAELQLPAPVGGAYT 116
pNDM-KN         FALSAGIDRKVKISVLRSDGTVVSTATSHLLGATDRITVGGKSYYGAELQLPAPVGGAYT 116
pUMNK88         FALSAGIDRKVKISVLRSDGTVVSTATSHLLGATDRITVGGKSYYGAELQLPAPVGGAYT 116
pCf587          FALSAGIDRKVKVSVRRSDGTVVSTATSHLLGATDRITVGGKSYYGAELQLAAPAAGSYT 116
                ************:** *********************************** **  * *.

pKAZ3           LRAEILASDGSSVQTDEYPLQVDTTAPSLANVTV----------KGEWNRALSDGTLLRG 166
pMS6198A        IRAEILASDGSTVQTDEYPLTVDVTPPTYSSLAPVYSNYGQVTSGDVWKLGLGGSE--DN 174
pKp55           IRAEILASDGSTVQTDEYPLTVDVTPPTYSSLAPVYSNYGQVTSGDVWKLGLGGSE--DN 178
pEc19           IRAEILASDGSTVQTDEYPLTVDVTPPTYSSLAPVYSNYGQVTSGDVWKLGLGGSE--DN 178
pAPEC1990       IRAEILASDGSTVQTDEYPLTVDVTPPTYSSLAPVYSNYGQVTSGDVWKLGLGGSE--DN 174
pNDM-KN         IRAEILASDGSTVQTDEYPLTVDVTPPTYSSLAPVYSNYGQVTSGDVWKLGLGGSE--DN 174
pUMNK88         IRAEILASDGSTVQTDEYPLTVDVTPPTYSSLAPVYSNYGQVTSGDVWKLGLGGSE--DN 174
pCf587          IRAEILASDGSTVQSDDYPLTVDVTPPKYTSLAPVYGTYGQVISGDVWKLGTGGAE--EN 174
                :**********:**:*:*** **.* *. :.::            . *: . ...    .

pKAZ3           PNRFSGIDVSASDAGSSVSSIQAYAVDSKGKRSPVVSVNYANGQGQL--LNWSTVFPN-- 222
pMS6198A        AFLLSGIS-----DESPIKGVKAKLYRQDGSLYKDVSVNYDDANGQARQSFQSGFFPASD 229
pKp55           AFLLSGIS-----DESPIKGVKAKLYRQDGSLYKDVSVNYDDANGQARQSFQSGFFPASD 233
pEc19           AFLLSGIS-----DESPIKGVKAKLYRQDGSLYKDVSVNYDDANGQARQSFQSGFFPASD 233
pAPEC1990       AFLLSGIS-----DESPIKGVKAKLYRQDGSLYKDVSVNYDDANGQARQSFQSGFFPASD 229
pNDM-KN         AFLLSGIS-----DESPIKGVKAKLYRQDGSLYKDVSVNYDDANGQARQSFQSGFFPASD 229
pUMNK88         AFLLSGIS-----DESPIKGVKAKLYRQDGSLYKDVSVNYDDANGQARQSFQSGFFPASD 229
pCf587          AFLISGIS-----DDSPVKEVKAKLYRQDGSLYKDVSVNYDDANKQARQYFESGFFPASD 229
                   :***.       * :. ::*    ..*.    ***** :.: *      * .**

pKAZ3           GEDLYTLHFEALDKAGNKGSIA-YPIAWDSVGAKSGENPEPVAVYDPKNPQASTFKVNGQ 281
pMS6198A        LDEVFTLQFEISDSAGNSYLSPRQKVMFDSI---TNAPSAPFGVYDPSST-----NNLGP 281
pKp55           LDEVFTLQFEISDSAGNSYLSPRQKVMFDSI---TNAPSAPFGVYDPSST-----NNLGP 285
pEc19           LDEVFTLQFEISDSAGNSYLSPRQKVMFDSI---TNAPSAPFGVYDPSST-----NNLGP 285
pAPEC1990       LDEVFTLQFEISDSAGNSYLSPRQKVMFDSI---TNAPSAPFGVYDPSST-----NNLGP 281
pNDM-KN         LDEVFTLQFEISDSAGNSYLSPRQKVMFDSI---TNAPSAPFGVYDPSST-----NNLGP 281
pUMNK88         LDEVFTLQFEISDSAGNSYLSPRQKVMFDSI---TNAPSAPFGVYDPSST-----NNLGP 281
pCf587          LDEVFTLQFQLSDSAGNSYLSPPQKVMFDNL---TNAPSSPFGVYDPASS-----STLGP 281
                 ::::**:*:  *.***.       : :*.:   :.    *..**** .      .  *

pKAZ3           ALSGFAPYQNGMTIYSDTYRVLYRIPKTNSYPSSPYGAQSGNWCDYKNCI--------EG 333
pMS6198A        GLTGFVAYTEGMTVKTNPIKLAWRVPRDN-------------WHEYREGGINMTNALGEM 328
pKp55           GLTGFVAYTEGMTVKTNPIKLAWRVPRDN-------------WHEYREGGINMTNALGEM 332
pEc19           GLTGFVAYTEGMTVKTNPIKLAWRVPRDN-------------WHEYREGGINMTNALGEM 332
pAPEC1990       GLTGFVAYTEGMTVKTNPIKLAWRVPRDN-------------WHEYREGGINMTNALGEM 328
pNDM-KN         GLTGFVAYTEGMTVKTNPIKLAWRVPRDN-------------WHEYREGGINMTNALGEM 328
pUMNK88         GLTGFVAYTEGMTVKTNPIKLAWRVPRDN-------------WHEYREGGINMTNALGEM 328
pCf587          GLSGFVAYKAGMTVKTNPIKLAWRVPRNN-------------WHEYREGGINMVNSLGEM 328
                .*:**. *  ***: ::  :: :*:*: *             * :*::          *

pKAZ3           NIIAEDGTYDYRQTQADVFQQHGTKTKSFIIYDWVMNS-L--GNTSVSVKADPSVSLAPI 390
pMS6198A        SKVGEDASYVYLVTTAPYGNTDGN------YWRWVNFGQWGGGGIAYNLTLSPSAPKSPR 382
pKp55           SKVGEDASYVYLVTTAPYGNTDGN------YWRWVNFGQWGGGGIAYNLTLSPSAPKSPR 386
pEc19           SKVGEDASYVYLVTTAPYGNTDGN------YWRWVNFGQWGGGGIAYNLTLSPSAPKSPR 386
pAPEC1990       SKVGEDASYVYLVTTAPYGNTDGN------YWRWVNFGQWGGGGIAYNLTLSPSAPKSPR 382
pNDM-KN         SKVGEDASYVYLVTTAPYGNTDGN------YWRWVNFGQWGGGGIAYNLTLSPSAPKSPR 382
pUMNK88         SKVGEDASYVYLVTTAPYGNTDGN------YWRWVNFGQWGGGGIAYNLTLSPSAPKSPR 382
pCf587          TKVGEDGNYVYLVTTAPYGNTDGN------YWRWVNFGQWGGGVIAYDLSLSPSAPQSPK 382
                . :.**..* *  * *   : .*.       : **  .    *  : .:. .**.  :*

pKAZ3           GKYVEYLRDDG-------QWVRGETINMSSVNHYKKLRFHVEPRPYAQELWGSWLPTTKI 443
pMS6198A        LLGVDYNYSDIGWSSFYRYWVNNSVLP----VTVSSIRVKVEPRPYVQTAVHRG--SC-- 434
pKp55           LLGVDYNYSDIGWSSFYRYWVNNSVLP----VTVSSIRVKVEPRPYVQTAVHRG--SC-- 438
pEc19           LLGVDYNYSDIGWSSFYRYWVNNSVLP----VTVSSIRVKVEPRPYVQTAVHRG--SC-- 438
```

```
pAPEC1990       LLGVDYNYSDIGWSSFYRYWVNNSVLP----VTVSSIRVKVEPRPYVQTAVHRG--SC--  434
pNDM-KN         LLGVDYNYSDIGWSSFYRYWVNNSVLP----VTVSSIRVKVEPRPYVQTAVHRG--SC--  434
pUMNK88         LLGVDYNYSDIGWSSFYRYWVNNSVLP----VTVSSIRVKVEPRPYVQTAVHRG--SC--  434
pCf587          LLGVDYNYSDIGWSSMYRYWVDSSVLP----VTVSSIRVKVEPRPYVQTAVHRG--SC--  434
                   *:*  .*         ** ...:        ..:*.:******.*        :

pKAZ3           PANASYAEVDTDISFSGRSCSWPGYWSRPEGKPELPSDR-------------IGAT--F  487
pMS6198A        -----------EIPVGQDSCVIANSFTMAKGTTGYVHDNATVFNPDKSLRSNPLWAEVNW  483
pKp55           -----------EIPVGQDSCVIANSFTMAKGTTGYVHDNATVFNPDKSLRSNPLWAEVNW  487
pEc19           -----------EIPVGQDSCVIANSFTMAKGTTGYVHDNATVFNPDKSLRSNPLWAEVNW  487
pAPEC1990       -----------EIPVGQDSCVIANSFTMAKGTTGYVHDNATVFNPDKSLRSNPLWAEVNW  483
pNDM-KN         -----------EIPVGQDSCVIANSFTMAKGTTGYVHDNATVFNPDKSLRSNPLWAEVNW  483
pUMNK88         -----------EIPVGQDSCVIANSFTMAKGTTGYVHDNATVFNPDKSLRSNPLWAEVNW  483
pCf587          -----------EVPVGQDSCVIANSFTMSKGTTGYIHDNATVFNADRSLSSNPLWAEVNW  483
                           :: ..  **   . ::  :*.     *.              : *   :

pKAZ3           CYDLNPPEIAGLERNGRIFKAHFREPDS---FDGWGV-NQWVISNNSSASAITSTGEERP  543
pMS6198A        NDQHYPQLSQQFDQNSKVFTLFVNQPGRGAYFDRLRLRSAWIEDSKG--NKLS-------  534
pKp55           NDQHYPQLSQQFDQNSKVFTLFVNQPGRGAYFDRLRLRSAWIEDSKG--NKLS-------  538
pEc19           NDQHYPQLSQQFDQNSKVFTLFVNQPGRGAYFDRLRLRSAWIEDSKG--NKLS-------  538
pAPEC1990       NDQHYPQLSQQFDQNSKVFTLFVNQPGRGAYFDRLRLRSAWIEDSKG--NKLS-------  534
pNDM-KN         NDQHYPQLSQQFDQNSKVFTLFVNQPGRGAYFDRLRLRSAWIEDSKG--NKLS-------  534
pUMNK88         NDQHYPQLSQQFDQNSKVFTLFVNQPGRGAYFDRLRLRSAWIEDSKG--NKLS-------  534
pCf587          NDQHYPQLSQQYDQSNKVFTLFVNQPGRGAYFDRLRLRSAWIEDSKG--NKLS-------  534
                  :  *      ::..::*. ...:*.    **   : . *: ..:.  . ::

pKAZ3           LSRTEVLRSSTNDWFFTFSAENLSEGTYTGISLVAKDAFGNEVKQVFTGSQYAMSIDNSA  603
pMS6198A        -PTGGLIANNWENYTYQWDLKTLPEGQYSLVAAA-EEMHG----PLTRQPMFQITSDRTP  588
pKp55           -PTGGLIANNWENYTYQWDLKTLPEGQYSLVAAA-EEMHG----PLTRQPMFQITSDRTP  592
pEc19           -PTGGLIANNWENYTYQWDLKTLPEGQYSLVAAA-EEMHG----PLTRQPMFQITSDRTP  592
pAPEC1990       -PTGGLIANNWENYTYQWDLKTLPEGQYSLVAAA-EEMHG----PLTRQPMFQITSDRTP  588
pNDM-KN         -PTGGLIANNWENYTYQWDLKTLPEGQYSLVAAA-EEMHG----PLTRQPMFQITSDRTP  588
pUMNK88         -PTGGLIANNWENYTYQWDLKTLPEGQYSLVAAA-EEMHG----PLTRQPMFQITSDRTP  588
pCf587          -PTGGLIANNWENYTYQWDLKTLPEGQYSLVAAA-EEMHG----PLTRQPMFQITSDKTA  588
                     :: .. ::: : :. :.* ** *: :: . :: .*     :     : :: *.:

pKAZ3           PTLTVSISDGAPIQSLDDVVITLTDTADPSPKLTSIALVGGPADDKVQLSWREESKGRFR  663
pMS6198A        PTMTLSVADGAAIQTLDDVVITLADAIDPSPKLTSIALVGGPANDKVQLSWREESKGRFR  648
pKp55           PTMTLSVADGAAIQTLDDVVITLADAIDPSPKLTSIALVGGPANDKVQLSWREESKGRFR  652
pEc19           PTMTLSVADGAAIQTLDDVVITLADAIDPSPKLTSIALVGGPANDKVQLSWREESKGRFR  652
pAPEC1990       PTMTLSVADGAAIQTLDDVVITLADAIDPSPKLTSIALVGGPANDKVQLSWREESKGRFR  648
pNDM-KN         PTMTLSVADGAAIQTLDDVVITLADAIDPSPKLTSIALVGGPANDKVQLSWREESKGRFR  648
pUMNK88         PTMTLSVADGAAIQTLDDVVITLADAIDPSPKLTSIALVGGPANDKVQLSWREESKGRFR  648
pCf587          PTLTISVADGAAIQTLDDVVITLADAIDPSPKLTSIALVGGPANDKVQLSWREESKGRFR  648
                **:*:*::*** **:********:*: ****************:****************

pKAZ3           LEYPVMFPSLKEGESYTLTVSGEDAQGNAVQKAVGFEYKPRQVMLADGMDGKVMVPAVTH  723
pMS6198A        LEYPVMFPSLKEGESYTLTVSGEDAQGNAVQKAVGFEYKPRQVMLADGMDGKVMVPAVTH  708
pKp55           LEYPVMFPSLKEGESYTLTVSGEDAQGNAVQKAVGFEYKPRQVMLADGMDGKVMVPAVTH  712
pEc19           LEYPVMFPSLKEGESYTLTVSGEDAQGNAVQKAVGFEYKPRQVMLADGMDGKVMVPAVTH  712
pAPEC1990       LEYPVMFPSLKEGESYTLTVSGEDAQGNAVQKAVGFEYKPRQVMLADGMDGKVMVPAVTH  708
pNDM-KN         LEYPVMFPSLKEGESYTLTVSGEDAQGNAVQKAVGFEYKPRQVMLADGMDGKVMVPAVTH  708
pUMNK88         LEYPVMFPSLKEGESYTLTVSGEDAQGNAVQKAVGFEYKPRQVMLADGMDGKVMVPAVTH  708
pCf587          LEYPVMFPSLKEGESYRLTVSGADAQGNAVKKVVGFEYKPRQVMLADGMDGKVMVPAVTH  708
                **************** ***** *******:*.***************************

pKAZ3           EFVHADGKRIIETKPLTLSDGAVVTGSYDVFATLRSDAKVPLVVNGVRIEPGQTMGIMSQ  783
pMS6198A        EFVHADGKRIIETKPLTLSDGAVVTGSYDVFATLRSDAKVPLVVNGVRIEPGQTMGIMSQ  768
pKp55           EFVHADGKRIIETKPLTLSDGAVVTGSYDVFATLRSDAKVPLVVNGVRIEPGQTMGIMSQ  772
pEc19           EFVHADGKRIIETKPLTLSDGAVVTGSYDVFATLRSDAKVPLVVNGVRIEPGQTMGIMSQ  772
pAPEC1990       EFVHADGKRIIETKPLTLSDGAVVTGSYDVFATLRSDAKVPLVVNGVRIEPGQTMGIMSQ  768
pNDM-KN         EFVHADGKRIIETKPLTLSDGAVVTGSYDVFATLRSDAKVPLVVNGVRIEPGQTMGIMSQ  768
pUMNK88         EFVHADGKRIIETKPLTLSDGAVVTGSYDVFATLRSDAKVPLVVNGVRIEPGQTMGIMSQ  768
pCf587          EFVHADGKRIIETKPLTLSDGAIVTGSYDVFATLRSDAKVPLVVNGVRIEPGQTMGIMSQ  768
                **********************:*************************************

pKAZ3           HDFGASGGRLSIPVKPAVPDVVGSSSLLVMTSAPNSPILVVDINTWKGTAKLSAESWTIR  843
pMS6198A        HDFGASGGRLSIPVKPAVPDVVGSSSLLVMTSAPNSPILVVDINTWKGTAKLSAESWTIR  828
pKp55           HDFGASGGRLSIPVKPAVPDVVGSSSLLVMTSAPNSPILVVDINTWKGTAKLSAESWTIR  832
pEc19           HDFGASGGRLSIPVKPAVPDVVGSSSLLVMTSAPNSPILVVDINTWKGTAKLSAESWTIR  832
pAPEC1990       HDFGASGGRLSIPVKPAVPDVVGSSSLLVMTSAPNSPILVVDINTWKGTAKLSAESWTIR  828
pNDM-KN         HDFGASGGRLSIPVKPAVPDVVGSSSLLVMTSAPNSPILVVDINTWKGTAKLSAESWTIR  828
pUMNK88         HDFGASGGRLSIPVKPAVPDVVGSSSLLVMTSAPNSPILVVDINTWKGTAKLSAESWTIR  828
pCf587          HDFGASGGRLSIPVKPAIPDVVGSSSLLVMTSAPNSPILVVDINTWKGAAKLSAESWTIR  828
                *****************:******************************:***********

pKAZ3           QVIDPVKIYALPESGVPCRFTTKEDVAMAADPIRDPVCLLQWDRTPDEAEQTTQDNNGMK  903
```

```
pMS6198A       QVIDPVKIYALPESGVPCRFTTKEDVAMAADPIRDPVCLLQWDRTPDEAEQTTQDNNGMK  888
pKp55          QVIDPVKIYALPESGVPCRFTTKEDVAMAADPIRDPVCLLQWDRTPDEAEQTTQDNNGMK  892
pEc19          QVIDPVKIYALPESGVPCRFTTKEDVAMAADPIRDPVCLLQWDRTPDEAEQTTQDNNGMK  892
pAPEC1990      QVIDPVKIYALPESGVPCRFTTKEDVAMAADPIRDPVCLLQWDRTPDEAEQTTQDNNGMK  888
pNDM-KN        QVIDPVKIYALPESGVPCRFTTKEDVAMAADPIRDPVCLLQWDRTPDEAEQTTQDNNGMK  888
pUMNK88        QVIDPVKIYALPESGVPCRFTTKEDVAMAADPIRDPVCLLQWDRTPDEAEQTTQDNNGMK  888
pCf587         QVIDPVKIYALPETGVPCRFTTKADVAMAADPIRDPVCLLQWDRTPDEAEQTTQDTNGMK  888
               *************:********* *******************************.****

pKAZ3          VAGLVGQAVSIGEQPVEYSLYLFSGDGSKVKVGSGSQNLTVTTAYGSVGYTPIDDIAQVN  963
pMS6198A       VAGLVGQAVSIGEQPVEYSLYLFSGDGSKVKVGSGSQNLTVTTAYGSVGYTPIDDIAQVN  948
pKp55          VAGLVGQAVSIGEQPVEYSLYLFSGDGSKVKVGSGSQNLTVTTAYGSVGYTPIDDIAQVN  952
pEc19          VAGLVGQAVSIGEQPVEYSLYLFSGDGSKVKVGSGSQNLTVTTAYGSVGYTPIDDIAQVN  952
pAPEC1990      VAGLVGQAVSIGEQPVEYSLYLFSGDGSKVKVGSGSQNLTVTTAYGSVGYTPIDDIAQVN  948
pNDM-KN        VAGLVGQAVSIGEQPVEYSLYLFSGDGSKVKVGSGSQNLTVTTAYGSVGYTPIDDIAQVN  948
pUMNK88        VAGLVGQAVSIGEQPVEYSLYLFSGDGSKVKVGSGSQNLTVTTAYGSVGYTPIDDIAQVN  948
pCf587         VAGLVGQAVSIGEQPVEYSLYLFSGDGSKVKVGSGSQNLTVTTAYGSVGYTPIDDIAQVN  948
               ************************************************************

pKAZ3          RVIEDFDVNFKQSKGPDCSITLSADRAKNEAANKAVGSASRTCLFEWQQIPDGLVQDPLS  1023
pMS6198A       RVIEDFDVNFKQSKGPDCSITLSADRAKKEAANKAVGSASRACLFEWQQIPDGLVQDPLS  1008
pKp55          RVIEDFDVNFKQSKGPDCSITLSADRAKKEAANKAVGSASRACLFEWQQIPDGLVQDPLS  1012
pEc19          RVIEDFDVNFKQSKGPDCSITLSADRAKKEAANKAVGSASRACLFEWQQIPDGLVQDPLS  1012
pAPEC1990      RVIEDFDVNFKQSKGPDCSITLSADRAKKEAANKAVGSASRACLFEWQQIPDGLVQDPLS  1008
pNDM-KN        RVIEDFDVNFKQSKGPDCSITLSADRAKKEAANKAVGSASRACLFEWQQIPDGLVQDPLS  1008
pUMNK88        RVIEDFDVNFKQSKGPDCSITLSADRAKKEAANKAVGSASRACLFEWQQIPDGLVQDPLS  1008
pCf587         RVIENFNVNLKQNKGPDCSITLSADRAKKEAASKGAGSASRTCLFEWQQIPDGLLQEQLS  1008
               ****:*:**:**.***************:***.*..*****:************:*: **

pKAZ3          ESPSLSGSLAENGDHPLGWRVSIFTRNGTRVTLNDETFNVEAVDPPAPTVELASDYNFKD  1083
pMS6198A       ESPSLSGSLASNGVHPLGWRVSIFTRNGTRVTLNDETFNVEAVDPPAPTVELASDYNFKD  1068
pKp55          ESPSLSGSLASNGVHPLGWRVSIFTRNGTRVTLNDETFNVEAVDPPAPTVELASDYNFKD  1072
pEc19          ESPSLSGSLASNGVHPLGWRVSIFTRNGTRVTLNDETFNVEAVDPPAPTVELASDYNFKD  1072
pAPEC1990      ESPSLSGSLASNGVHPLGWRVSIFTRNGTRVTLNDETFNVEAVDPPAPTVELASDYNFKD  1068
pNDM-KN        ESPSLSGSLASNGVHPLGWRVSIFTRNGTRVTLNDETFNVEAVDPPAPTVELASDYNFKD  1068
pUMNK88        ESPSLSGSLASNGVHPLGWRVSIFTRNGTRVTLNDETFNVEAVDPPAPTVELASDYNFKD  1068
pCf587         ESPSLSGSLASNGNHPLGWRVSIFTRNGTRVTLNDQTFNIEAVDPPAPTVELSSKFNFKD  1068
               **********.** *********************:***:************:*.:****

pKAZ3          NIYLVPMTGNYLGDAIINSERADLDIAISRNSDVLESETFTPGWGATNKVYRRINTDERA  1143
pMS6198A       NIYLVPMTGNYLGDAIINSERADLDIAISRNSDVLESETFTPGWGATNKVYRRINTDERA  1128
pKp55          NIYLVPMTGNYLGDAIINSERADLDIAISRNSDVLESETFTPGWGATNKVYRRINTDERA  1132
pEc19          NIYLVPMTGNYLGDAIINSERADLDIAISRNSDVLESETFTPGWGATNKVYRRINTDERA  1132
pAPEC1990      NIYLVPMTGNYLGDAIINSERADLDIAISRNSDVLESETFTPGWGATNKVYRRINTDERA  1128
pNDM-KN        NIYLVPMTGNYLGDAIINSERADLDIAISRNSDVLESETFTPGWGATNKVYRRINTDERA  1128
pUMNK88        NIYLVPMTGNYLGDAIINSERADLDIAISRNSDVLESETFTPGWGATNKVYRRINTDERA  1128
pCf587         NIYMVPMTGNYLGDAIISSERADLDIAISRNSGVLESETFAPGWGATNKVYRRINTDERA  1128
               ***:*************.**************.*******:*******************

pKAZ3          LWEETTYKVNAAYNKVPDVKTEVVYRAISAPSDSIRPIVEVKGDTAIDTQALPVRVLIRD  1203
pMS6198A       LWEETTYKVNAAYNKVPDVKTEVVYRAISAPSDSIRPIVEVKGDTAIDTQALPVRVLIRD  1188
pKp55          LWEETTYKVNAAYNKVPDVKTEVVYRAISAPSDSIRPIVEVKGDTAIDTQALPVRVLIRD  1192
pEc19          LWEETTYKVNAAYNKVPDVKTEVVYRAISAPSDSIRPIVEVKGDTAIDTQALPVRVLIRD  1192
pAPEC1990      LWEETTYKVNAAYNKVPDVKTEVVYRAISAPSDSIRPIVEVKGDTAIDTQALPVRVLIRD  1188
pNDM-KN        LWEETTYKVNAAYNKVPDVKTEVVYRAISAPSDSIRPIVEVKGDTAIDTQALPVRVLIRD  1188
pUMNK88        LWEETTYKVNAAYNKVPDVKTEVVYRAISAPSDSIRPIVEVKGDTAIDTQALPVRVLIRD  1188
pCf587         LWEETTYKVNAAYNKVPDVKTEAIYRAIAAPSDSIRPVVEVDGDNAIDTQALPVRVLIRD  1188
               **********************.:****:********:***.**.***************

pKAZ3          QYKPDGDYDANTMGVWKVRLIQQKAYNETVALTDYAEASNGEAQFSVDLSGVDTSSVRIA  1263
pMS6198A       QYKPDGDYDANTMGVWKVRLIQQKAYNETVALTDYAEASNGEAQFSVDLSGVDTSSVRIA  1248
pKp55          QYKPDGDYDANTMGVWKVRLIQQKAYNETVALTDYAEASNGEAQFSVDLSGVDTSSVRIA  1252
pEc19          QYKPDGDYDANTMGVWKVRLIQQKAYNETVALTDYAEASNGEAQFSVDLSGVDTSSVRIA  1252
pAPEC1990      QYKPDGDYDANTMGVWKVRLIQQKAYNETVALTDYAEASNGEAQFSVDLSGVDTSSVRIA  1248
pNDM-KN        QYKPDGDYDANTMGVWKVRLIQQKAYNETVALTDYAEASNGEAQFSVDLSGVDTSSVRIA  1248
pUMNK88        QYKPDGDYDANTMGVWKVRLIQQKAYNETVALTDYAEASNGEAQFSVDLSGVDTSSVRIA  1248
pCf587         QYKPDGGYDANTMGVWKVRLIQQKAYNETVALTDYAEASNGEAQFSVDLSGVDTSSVRIA  1248
               ******.*****************************************************

pKAZ3          AEAVLESPVEGYNRTELSIRPAFLTVLRGGAIGAGVEARKLSGEAPFTAVFKLSLDDRQD  1323
pMS6198A       AEAVLESPVEGYNRTELSIRPAFLTVLRGGAIGAGVEARKLSGEAPFTAVFKLSLDDRQD  1308
pKp55          AEAVLESPVEGYNRTELSIRPAFLTVLRGGAIGAGVEARKLSGEAPFTAVFKLSLDDRQD  1312
pEc19          AEAVLESPVEGYNRTELSIRPAFLTVLRGGAIGAGVEARKLSGEAPFTAVFKLSLDDRQD  1312
pAPEC1990      AEAVLESPVEGYNRTELSIRPAFLTVLRGGAIGAGVEARKLSGEAPFTAVFKLSLDDRQD  1308
pNDM-KN        AEAVLESPVEGYNRTELSIRPAFLTVLRGGAIGAGVEARKLSGEAPFTAVFKLSLDDRQD  1308
pUMNK88        AEAVLESPVEGYNRTELSIRPAFLTVLRGGAIGAGVEARKLSGEAPFTAVFKLSLDDRQD  1308
pCf587         AEAVLDSPVEGYNRTELSIRPAFLTVLRGGAIAAGVEARKLSGEAPFTAVFKLALDNRQD  1308
```

```
                    *****:**************************.********************:**:***

pKAZ3               LRATGQVVWETSKDDGKTWEQFIPEDRYKYQLVKTFDKGEYQVRAKVVNVNSGAEKYTEA 1383
pMS6198A            LRATGQVVWETSKDDGKTWEQFIPEDRYKYQLVKTFDKGEYQVRAKVVNVNSGAEKYTEA 1368
pKp55               LRATGQVVWETSKDDGKTWEQFIPEDRYKYQLVKTFDKGEYQVRAKVVNVNSGAEKYTEA 1372
pEc19               LRATGQVVWETSKDDGKTWEQFIPEDRYKYQLVKTFDKGEYQVRAKVVNVNSGAEKYTEA 1372
pAPEC1990           LRATGQVVWETSKDDGKTWEQFIPEDRYKYQLVKTFDKGEYQVRAKVVNVNSGAEKYTEA 1368
pNDM-KN             LRATGQVVWETSKDDGKTWEQFIPEDRYKYQLVKTFDKGEYQVRAKVVNVNSGAEKYTEA 1368
pUMNK88             LRATGQVVWETSKDDGKTWEQFIPEDRYKYQLVKTFDKGEYQVRAKVVNVNSGAEKYTEA 1368
pCf587              LRATGQVVWETTKDDGKTWEQFIPEERYKYQLVKTFDKGEYQVRAKVVNVNSGAEKYTEA 1368
                    ***********:*************:**********************************

pKAZ3               VSVVAYDKPDIAVIGPTTLFVGSEGKYTANLTLNDEPISGGNAIVEWSTDGGKTYAQTGD 1443
pMS6198A            VSVVAYDKPDIAVIGPTTLFVGSEGKYTANLTLNDEPISGGNAIVEWSTDGGKTYAQTGD 1428
pKp55               VSVVAYDKPDIAVIGPTTLFVGSEGKYTANLTLNDEPISGGNAIVEWSTDGGKTYAQTGD 1432
pEc19               VSVVAYDKPDIAVIGPTTLFVGSEGKYTANLTLNDEPISGGNAIVEWSTDGGKTYAQTGD 1432
pAPEC1990           VSVVAYDKPDIAVIGPTTLFVGSEGKYTANLTLNDEPISGGNAIVEWSTDGGKTYAQTGD 1428
pNDM-KN             VSVVAYDKPDIAVIGPTTLFVGSEGKYTANLTLNDEPISGGNAIVEWSTDGGKTYAQTGD 1428
pUMNK88             VSVVAYDKPDIAVIGPTTLFVGSEGKYTANLTLNDEPISGGNAIVEWSTDGGKTYAQTGD 1428
pCf587              VSVVAYDKPDIAVVGPTTLFVGSEGKYTASLALNDEPITDGNAVVEWSTDGGKTYSHKGN 1428
                    *************:***************.*:******:.***:***********::.*:

pKAZ3               SITLSSDEETRYRLWARVRSATAPADDGYAYEVAKTAVDFRAVKAPRPYVTGPRVIETGK 1503
pMS6198A            SITLSSDEETRYRLWARVRSATAPADDGYAYEVAKTAVDFRAVKAPRPYVTGPRVIETGK 1488
pKp55               SITLSSDEETRYRLWARVRSATAPADDGYAYEVAKTAVDFRAVKAPRPYVTGPRVIETGK 1492
pEc19               SITLSSDEETRYRLWARVRSATAPADDGYAYEVAKTAVDFRAVKAPRPYVTGPRVIETGK 1492
pAPEC1990           SITLSSDEETRYRLWARVRSATAPADDGYAYEVAKTAVDFRAVKAPRPYVTGPRVIETGK 1488
pNDM-KN             SITLSSDEETRYRLWARVRSATAPADDGYAYEVAKTAVDFRAVKAPRPYVTGPRVIETGK 1488
pUMNK88             SITLSSDEETRYRLWARVRSATAPADDGYAYEVAKTAVDFRAVKAPRPYVTGPRVIETGK 1488
pCf587              SITLSSDEETRYRLWARVRSANAPADDDYAYDVAKTAVDFRAVKAPRPYVVGPQVIETGK 1488
                    *********************.*****.***:******************.**:******

pKAZ3               KYVFKAETSLPYRGMDVKLNGFFTLPDGSIVQGDTAEYVPSDTDLNQATVETKYTTWIEG 1563
pMS6198A            KYVFKAETSLPYRGMDVKLNGFFTLPDGSIVQGDTAEYEPSDTDLNQATVETKYTTWIEG 1548
pKp55               KYVFKAETSLPYRGMDVKLNGFFTLPDGSIVQGDTAEYEPSDTDLNQATVETKYTTWIEG 1552
pEc19               KYVFKAETSLPYRGMDVKLNGFFTLPDGSIVQGDTAEYEPSDTDLNQATVETKYTTWIEG 1552
pAPEC1990           KYVFKAETSLPYRGMDVKLNGFFTLPDGSIVQGDTAEYEPSDTDLNQATVETKYTTWIEG 1548
pNDM-KN             KYVFKAETSLPYRGMDVKLNGFFTLPDGSIVQGDTAEYEPSDTDLNQATVETKYTTWIEG 1548
pUMNK88             KYVFKAETSLPYRGMDVKLNGFFTLPDGSIVQGDTAEYEPSDTDLNQATVETKYTTWIEG 1548
pCf587              KYVFSAKTSLPYRGMDVKLNGFFTLPDGSIVEGDTAEYVPSDNDLNQATVETKYTTWIEG 1548
                    ****.*:************************:****** ***.*****************

pKAZ3               YRDQGAEASHSLRSRVWQYVWPSFGMQVRKNADVAPATITASVRPIAFNGKLEEPTYEWE 1623
pMS6198A            YRDQGAEASHSLRSRVWQYVWPSFGMQVRKNADVAPATITASVRPIAFNGKLEEPTYEWE 1608
pKp55               YRDQGAEASHSLRSRVWQYVWPSFGMQVRKNADVAPATITASVRPIAFNGKLEEPTYEWE 1612
pEc19               YRDQGAEASHSLRSRVWQYVWPSFGMQVRKNADVAPATITASVRPIAFNGKLEEPTYEWE 1612
pAPEC1990           YRDQGAEASHSLRSRVWQYVWPSFGMQVRKNADVAPATITASVRPIAFNGKLEEPTYEWE 1608
pNDM-KN             YRDQGAEASHSLRSRVWQYVWPSFGMQVRKNADVAPATITASVRPIAFNGKLEEPTYEWE 1608
pUMNK88             YRDQGAEASHSLRSRVWQYVWPSFGMQVRKNADVAPATITASVRPIAFNGKLEEPTYEWE 1608
pCf587              YRDKGAEASHSLRSRVWQYVWPGFAMQVRKSADAAPATITASVRPIAFNGKLEEPTYEWE 1608
                    ***:******************.*.*****.**.**************************

pKAZ3               LPEGAVIQDQRQDIVRSFVINEPGDYNIKVTVRDARGHETVIEQPLKIGQAEPYAIDLQY 1683
pMS6198A            LPEGAVIQDQRQDIVRSFVINEPGDYNIKVTVRDARGHETVIEQPLKIGQAAPYAIDLQY 1668
pKp55               LPEGAVIQDQRQDIVRSFVINEPGDYNIKVTVRDARGHETVIEQPLKIGQAAPYAIDLQY 1672
pEc19               LPEGAVIQDQRQDIVRSFVINEPGDYNIKVTVRDARGHETVIEQPLKIGQAAPYAIDLQY 1672
pAPEC1990           LPEGAVIQDQRQDIVRSFVINEPGDYNIKVTVRDARGHETVIEQPLKIGQAAPYAIDLQY 1668
pNDM-KN             LPEGAVIQDQRQDIVRSFVINEPGDYNIKVTVRDARGHETVIEQPLKIGQAAPYAIDLQY 1668
pUMNK88             LPEGAVIQDQRQDIVRSFVINEPGDYNIKVTVRDARGHETVIEQPLKIGQAAPYAIDLQY 1668
pCf587              LPEGAVIQDKKQDIVRSFVVNEPGDYNIKVTVRDARGHEAVIEKSFKIDQSKPYAIDLQY 1668
                    *********::********:*******************:***: :**.*: ********

pKAZ3               SGSNKYEREPLDVLLRPYISGGHPRDRISTRVYSVDGTPLESSGYYGRATLGAGEHSIKL 1743
pMS6198A            SGSNKYEREPLDVLLRPYISGGHPRDRISTRVYSVDGTPLESSGYYGRATLGAGEHSIKL 1728
pKp55               SGSNKYEREPLDVLLRPYISGGHPRDRISTRVYSVDGTPLESSGYYGRATLGAGEHSIKL 1732
pEc19               SGSNKYEREPLDVLLRPYISGGHPRDRISTRVYSVDGTPLESSGYYGRATLGAGEHSIKL 1732
pAPEC1990           SGSNKYEREPLDVLLRPYISGGHPRDRISTRVYSVDGTPLESSGYYGRATLGAGEHSIKL 1728
pNDM-KN             SGSNKYEREPLDVLLRPYISGGHPRDRISTRVYSVDGTPLESSGYYGRATLGAGEHSIKL 1728
pUMNK88             SGSNKYEREPLDVLLRPYISGGHPRDRISTRVYSVDGTPLESSGYYGRATLGAGEHSIKL 1728
pCf587              SGSNKYEREPLDVLLRPYISGGHPRDRILTHVYSVDGTPLENSGYYGKATLRAGEHSIKL 1728
                    **************************** *:**********.*****:*** ********

pKAZ3               KITSEMGHEAEGEVNINVAENKLPACSLSSRETVGSWIVYANCEDTDGRMKSYEWTIAGE 1803
pMS6198A            KITSEMGHEAEGEVNINVAENKLPACSLSSRETVGSWIVYANCEDTDGRMKSYEWTIAGE 1788
pKp55               KITSEMGHEAEGEVNINVAENKLPACSLSSRETVGSWIVYANCEDTDGRMKSYEWTIAGE 1792
pEc19               KITSEMGHEAEGEVNINVAENKLPACSLSSRETVGSWIVYANCEDTDGRMKSYEWTIAGE 1792
pAPEC1990           KITSEMGHEAEGEVNINVAENKLPACSLSSRETVGSWIVYANCEDTDGRMKSYEWTIAGE 1788
```

```
pNDM-KN        KITSEMGHEAEGEVNINVAENKLPACSLSSRETVGSWIVYANCEDTDGRMKSYEWTIAGE 1788
pUMNK88        KITSEMGHEAEGEVNINVAENKLPACSLSSRETVGSWIVYANCEDTDGRMKSYEWTIAGE 1788
pCf587         KITSEMGHEAEGEVSINVAENKLPVCSLKSRETVGSWIVYANCEDTDGRMKSYEWTIAGE 1788
               **************.*********.***.*******************************

pKAZ3          LQSISSDRVTISKGTYETMPTISLVGVDDSGGKSEAVTMN  1843
pMS6198A       LQSISSDRVTISKGTYETMPTISLVGVDDSGGKSEAVTMN  1828
pKp55          LQSISSDRVTISKGTYETMPTISLVGVDDSGGKSEAVTMN  1832
pEc19          LQSISSDRVTISKGTYETMPTISLVGVDDSGGKSEAVTMN  1832
pAPEC1990      LQSISSDRVTISKGTYETMPTISLVGVDDSGGKSEAVTMN  1828
pNDM-KN        LQSISSDRVTISKGTYETMPTISLVGVDDSGGKSEAVTMN  1828
pUMNK88        LQSISSDRVTISKGTYETMPTISLVGVDDSGGKSEAVTMN  1828
pCf587         LKSISSDRVTISKGPNKTMPTISLVGVDDSGGRSEAVTMN  1828
               *:************  :***************:*******
```
