# Supplementary figures and images for: Roles of Proteins Containing Immunoglobulin-Like Domains in the Conjugation of Bacterial Plasmids
Source: mSphere. 2022 Jan 5;7(1):e00978-21. doi: 10.1128/msphere.00978-21 (PMC8730810; doi:10.1128/msphere.00978-21)

IncA/C

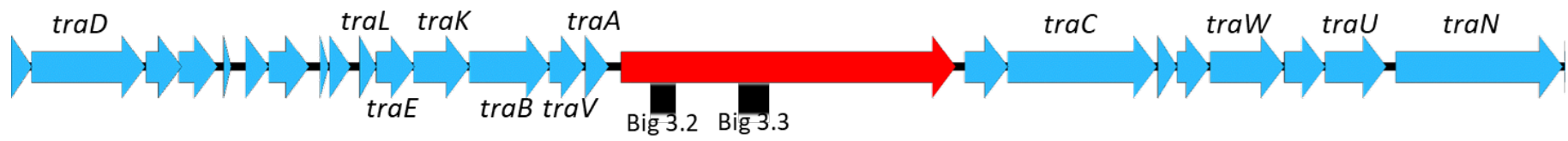

Supplement: FIG S2 [file msphere.00978-21-sf002.pdf]

IncP

Pilin-related protein

Big 1

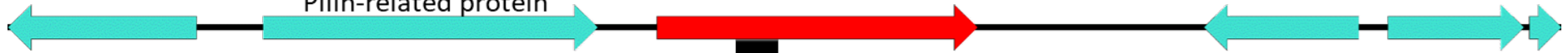

Supplement: FIG S3 [file msphere.00978-21-sf003.pdf]

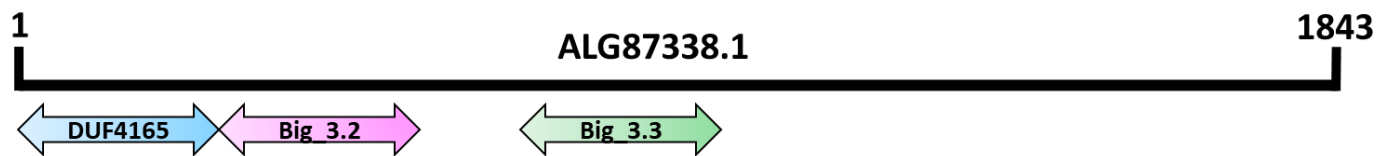

Supplement: FIG S5 [file msphere.00978-21-sf005.pdf]
