# Supplementary material for: Roles of Proteins Containing Immunoglobulin-Like Domains in the Conjugation of Bacterial Plasmids
Source: mSphere. 2022 Jan 5;7(1):e00978-21. doi: 10.1128/msphere.00978-21 (PMC8730810; doi:10.1128/msphere.00978-21)
Supplement: FIG S4 [file msphere.00978-21-sf004.pdf]

```
CKA44           --------------------------------------------------------MGLT    4
HMPREF1223      MFANLKALAVAGAFFLMSVTTTVSADSWKPIRSGSSSSGWQKVVCDRSGNGWRSCNMGLT    60
pOZ176          --------------------------------------------------------MGLT    4
pJB37           MFANLKALAVAGAFFLMSVTTTVSADSWKPIRSGSSSSGWQKVVCDRSGNGWRSCNMGLT    60
pPWIS1          --------------------------------------------------------MGLT    4
pAPA25          --------------------------------------------------------MGLT    4
pNK546KPC       MFANLKALAVAGAFFLMSVTTTVSADSWKPIRSGSSSSGWQKVVCDRSGNGWRSCNMGLT    60
RN02            --------------------------------------------------------MGLT    4
                                                                        ****

CKA44           IVIQATPGSLAALGEKATLVATVQDYDGNNAGRGVVINWTTSDGGLSAATTTTDANGQTS    64
HMPREF1223      IVIQATPGSLAALGEKATLVATVQDYDGNNAGRGVVINWTTSDGGLSAATTTTDANGQTS    120
pOZ176          IVIQATPGSLAALGEKATLVATVQDYDGNNAGRGVVINWTTSDGGLSAATTTTDANGQTS    64
pJB37           IVIQATPGSLAALGEKATLVATVQDYDGNNAGRGVVINWTTSDGGLSAATTTTDANGQTS    120
pPWIS1          IVIQATPGSLAALGEKATLVATVQDYDGNNAGRGVVINWTTSDGGLSAATTTTDANGQTS    64
pAPA25          IVIQATPGSLAALGEKATLVATVQDYDGNNAGRGVVINWTTSDGGLSAATTTTDANGQTS    64
pNK546KPC       IVIQATPGSLAALGEKATLVATVQDYDGNNAGRGVVINWTTSDGGLSAATTTTDANGQTS    120
RN02            IVIQATPGSLAALGEKATLVATVQDYDGNNAGRGVVINWTTSDGGLSAATTTTDANGQTS    64
                ************************************************************

CKA44           VVLTSSKTIGGATVSATSPAEGGTGQITVPFTDKWVSTSAMYSAWQDSGAPYSCSAWSPD    124
HMPREF1223      VVLTSSKTIGGATVSATSPAEGGTGQITVPFTDKWVSTSAIYSAWQDSGAPYSCSAWSPD    180
pOZ176          VVLTSSKTIGGATVSATSPAEGGTGQITVPFTDKWVSTSAMYSAWQDSGAPYSCSAWSPD    124
pJB37           VVLTSSKTIGGATVSATSPAEGGTGQITVPFTDKWVSTSAMYSAWQDSGAPYSCSAWSPD    180
pPWIS1          VVLTSSKTIGGATVSATSPAEGGTGQITVPFTDKWVSTSAMYSAWQDSGAPYSCSAWSPD    124
pAPA25          VVLTSSKTIGGATVSATSPAEGGTGQITVPFTDKWVSTSAMYSAWQDSGAPYSCSAWSPD    124
pNK546KPC       VVLTSSKTIGGATVSATSPAEGGTGQITVPFTDKWVSTSAMYSAWQDSGAPYSCSAWSPD    180
RN02            VVLTSSKTIGGATVSATSPAEGGTGQITVPFTDKWVSTSAMYSAWQDSGAPYSCSAWSPD    124
                ****************************************:*******************

CKA44           VSTINQGTSFTQSAVCYQNQIAYQQNREVSLVTGQVRNVGGVIPLYQTVQAARSQQAVGT    184
HMPREF1223      ASTINQGTSFTQSAVCYQNQIAYQQNREVSLVTGQVRNVGGVIPLYQTVQAARSQQAVGT    240
pOZ176          ASTINQGTSFTQSAVCYQNQIAYQQNREVSLVTGQVRNVGGVIPLYQTVQAARSQQAVGT    184
pJB37           ASTINQGTSFTQSAVCYQNQIAYQQNREVSLVTGQVRNVGGVIPLYQTVQAARSQQAVGT    240
pPWIS1          ASTINQGTSFTQSAVCYQNQIAYQQNREVSLVTGQVRNVGGVIPLYQTVQAARSQQAVGT    184
pAPA25          ASTINQGTSFTQSAVCYQNQIAYQQNREVSLVTGQVRNVGGVIPLYQTVQAARSQQAVGT    184
pNK546KPC       ASTINQGTSFTQSAVCYQNQIAYQQNREVSLVTGQVRNVGGVIPLYQTVQAARSQQAVGT    240
RN02            ASTINQGTSFTQSAVCYQNQIAYQQNREVSLVTGQVRNVGGVIPLYQTVQAARSQQAVGT    184
                .***********************************************************

CKA44           KQSTPSCAWSSFTKNGVYATGWDHGVSNTGGPKQGYRLYLGQYIGEVANATDSFAYNGRI    244
HMPREF1223      KQSTPSCAWSSFTKNGVYATGWDHGVSNTGGPKQGYRLYLGQYIGEVANATDSFAYNGRI    300
pOZ176          KQSTPSCAWSSFTKNGVYATGWDHGVSNTGGPKQGYRLYLGQYIGEVANATDSFAYNGRI    244
pJB37           KQSTPSCAWSSFTKNGVYATGWDHGVSNTGGPKQGYRLYLGQYIGEVANATDSFAYNGRI    300
pPWIS1          KQSTPSCAWSSFTKNGVYATGWDHGVSNTGGPKQGYRLYLGQYIGEVANATDSFAYNGRI    244
pAPA25          KQSTPSCAWSSFTKNGVYATGWDHGVSNTGGPKQGYRLYLGQYIGEVANATDSFAYNGRI    244
pNK546KPC       KQSTPSCAWSSFTKNGVYATGWDHGVSNTGGPKQGYRLYLGQYIGEVANATDSFAYNGRI    300
RN02            KQSTPSCAWSSFTKNGVYATGWDHGVSNTGGPKQGYRLYLGQYIGEVANATDSFAYNGRI    244
                ************************************************************

CKA44           YTIGKFRQSTCLGKNCASSREEYEACSVPQ    274
HMPREF1223      YTIGKFRQSTCLGKNCASSREEYEACSVPQ    330
pOZ176          YTIGKFRQSTCLGKNCASSREEYEACSVPQ    274
pJB37           YTIGKFRQSTCLGKNCASSREEYEACSVPQ    330
pPWIS1          YTIGKFRQSTCLGKNCASSREEYEACSVPQ    274
pAPA25          YTIGKFRQSTCLGKNCASSREEYEACSVPQ    274
pNK546KPC       YTIGKFRQSTCLGKNCASSREEYEACSVPQ    330
RN02            YTIGKFRQSTCLGKNCASSREEYEACSVPQ    274
                ******************************
```
