# Supplementary material for: Roles of Proteins Containing Immunoglobulin-Like Domains in the Conjugation of Bacterial Plasmids
Source: mSphere. 2022 Jan 5;7(1):e00978-21. doi: 10.1128/msphere.00978-21 (PMC8730810; doi:10.1128/msphere.00978-21)
Supplement: TABLE S3 [file msphere.00978-21-st003.docx]

| **Strains** | **Relevant characteristics** | **Reference/Source** |
| --- | --- | --- |
| SL1344 | *rspL, hisG* | (1) |
| SL1344ibplac | *ibpA_420_::lacZ.* Km^r^ | (2) |
| SL1344 Δ*fliC* Δ*fljB* | *fliC*::FRT *fljB*::FRT | This work |
| **Plasmids** | **Relevant characteristics** | **Reference/Source** |
| R27 | IncHI1, Tc^r^ | (3) |
| pKAZ3 | IncA/C, Cb^r^, Tcr | Dr. Álvaro San Millán |
| R27 Δ*rsp2* | R27 *rsp2*::FRT, Tc^r^ | This work |
| R27 RSP-Flag | R27 *rsp*::Flag, Tc^r^, Km^r^ | (4) |
| R27 RSP2-Flag | R27 *rsp2*::Flag, Tc^r^, Km^r^ | This work |
| R27 Δ*rsp* | R27 *rsp*::FRT, Tc^r^ | (4) |
| R27 Δ*rsp* RSP2-Flag | R27 *rsp*::FRT, *rsp2*::Flag, Tc^r^, Km^r^ | This work |
| R27 Δ*trhC* | R27 *trhC*::FRT, Tc^r^ | (4) |
| R27 Δ*trhC* RSP-Flag | R27 *trhC*::Cm, *rsp*::Flag. Cm^r^, Km^r^, Tc^r^ | (4) |
| R27 Δ*trhC* RSP2-Flag | R27 *trhC*::Cm, *rsp2*::Flag. Cm^r^, Km^r^, Tc^r^ | This work |
| R27 Δ*trhH* | R27 *trhH*::FRT, Tc^r^ | This work |
| R27 Δ*trhH* RSP-Flag | R27 *trhH*::FRT, *rsp*::Flag. Km^r^, Tc^r^ | This work |
| R27 Δ*trhH* RSP2-Flag | R27 *trhH*::FRT, *rsp2*::Flag. Km^r^, Tc^r^ | This work |
| R27 Δ*trhA* | R27 *trhA*::FRT, Tc^r^ | This work |
| R27 Δ*trhA* RSP-Flag | R27 *trhA*::FRT, *rsp*::Flag. Km^r^, Tc^r^ | This work |
| R27 Δ*trhA* RSP2-Flag | R27 *trhA*::FRT, *rsp2*::Flag. Km^r^, Tc^r^ | This work |
| pLG338-*rsp* | pLG338-30 + *rsp* from R27 | (4) |
| pLG338-30 | ori_p_SC101, Cb^r^ | (5) |
| pLG338-*rsp2* | pLG338-30 + *rsp2* from R27 | This work |
| pBR322-*trhC* | pBR322 + *trhC* from R27 | (4) |
| pKD4 | *bla* FRT *ahp* FRT PS1 PS2 oriR6K Km^r^, Cb^r^ | (6) |
| pKD3 | *bla* FRT *cat* FRT PS1 PS2 oriR6K Cm^r^, Cb^r^ | (6) |
| pSUB11 | Flag- and Km^r^-coding template vector | (7) |
| pKD46 | *oriR101, repA101 (ts), AraBp-gam-bet-exo* | (6) |
| pKD46-Km^R^ | *oriR101, repA101 (ts), AraBp-gam-bet-exo*, Km^r^ | This work |
| pKAZ3 ΔALG87338.1 | pKAZ3 ALG87338.1:: Cm, Cm^r^, Tc^r^ | This work |

**Supporting References**

1. Hoiseth SK, Stocker BAD. 1981. Aromatic-dependent *Salmonella* Typhimurium are non-virulent and effective as live vaccines. Nature 291:238–239.

2. Hüttener M, Prieto A, Aznar S, Dietrich M, Paytubi S, Juárez A. 2018. Tetracycline alters gene expression in *Salmonella* strains that harbor the Tn10 transposon. Environmental Microbiology Reports 10:202–209.

3. Grindley NDF, Grindley JN, Anderson ES. 1972. R factor compatibility groups. MGG Molecular & General Genetics 119:287–297.

4. Hüttener M, Prieto A, Aznar S, Bernabeu M, Glaría E, Valledor AF, Paytubi S, Merino S, Tomás J, Juárez A. 2019. Expression of a novel class of bacterial Ig-like proteins is required for IncHI plasmid conjugation. PLoS Genetics 15:e1008399.

5. Cunningham TP, Montelaro RC, Rushlow KE. 1993. Lentivirus envelope sequences and pro viral genomes are stabilized in *Escherichia coli* when cloned in low-copy-number plasmid vectors. Gene 124:93–98.

6. Datsenko KA, Wanner BL. 2000. One-step inactivation of chromosomal genes in *Escherichia coli* K-12 using PCR products. Proceedings of the National Academy of Sciences of the United States of America 97:6640–6645.

7. Uzzau S, Figueroa-Bossi N, Rubino S, Bossi L. 2001. Epitope tagging of chromosomal genes in *Salmonella*. Proceedings of the National Academy of Sciences of the United States of America 98:15264–15269.
