# Supplementary material for: Roles of Proteins Containing Immunoglobulin-Like Domains in the Conjugation of Bacterial Plasmids
Source: mSphere. 2022 Jan 5;7(1):e00978-21. doi: 10.1128/msphere.00978-21 (PMC8730810; doi:10.1128/msphere.00978-21)
Supplement: TABLE S4 [file msphere.00978-21-st004.docx]

| **Name** | **Sequence 5’ - 3’** | **Use** |
| --- | --- | --- |
| **fliC_SL1344_P1** | CATCAAGTTGTAATTGATAAGGAAAAGATCATGGCAGTGTAGGCTGGAGCTGCTTC | ***fliC* deletion** |
| **fliC_SL1344_P2** | GTACCACGTGTCGGTGAATCAATCGCCGGATTAACGCATATGAATATCCTCCTTAGT | ***fliC* deletion** |
| **fliC_SL1344_P1Up** | CGTTCTTTGTCAGGTCTGTCAACAACTGG | ***fliC* deletion confirmation** |
| **fliC_SL1344_P2down** | GCAGGCAAGACTCAGAGAGTTACGCACC | ***fliC* deletion confirmation** |
| **fljB_SL1344_P1** | GCTTTATCAAAAACCTTCCAAAAGGAAAATTTTATGGCAGTGTAGGCTGGAGCTGCTTC | ***fljB* deletion** |
| **fljB_SL1344_P2** | AATTCACGGGGCTGAATAAAACGAAATAAATTAACGCATATGAATATCCTCCTTAGT | ***fljB* deletion** |
| **fljB_SL1344_P1up** | CGCCACCAGGTTTTTCACGCT | ***fljB* deletion confirmation** |
| **fljB_SL1344_P2down** | CCTGTCGTTTTGCCAGTCAAAACCTGTCC | ***fljB* deletion confirmation** |
| **RSP2_R27_P1** | GGTAAAATTTTCTGGCCAACCAGGAGAACCGAAATGAAATTTGTGTAGGCTGGAGCTGCTTC | ***rsp2* deletion** |
| **RSP2_R27_P2** | AAGCCCCGTAATACGGGGCCGGTTCGGAGGCAGTTACTGGATCATATGAATATCCTCCTTAGT | ***rsp2* deletion** |
| **RSP2_R27_P1up** | CAGGCCTGCCGATTAAATCTG | ***rsp2* deletion confirmation** |
| **RSP2_R27_P2down** | CTGGCTCTGCATTCGAATTTC | ***rsp2* deletion confirmation** |
| **RSP2_R27_3xP1** | CGGTGAATCAAGCTTCATGATCGAGCTGCCGCAAATCCAGGACTACAAAGACCATGACGG | **RSP2-Flag** |
| **RSP2_R27_3xP2** | AAGCCCCGTAATACGGGGCCGGTTCGGAGGCAGTTACTGGCATATGAATATCCTCCTTAG | **RSP2-Flag** |
| **RSP2_R27_3xP1up** | GCGGTAGATGAAGCCGGTAAT | **RSP2-Flag confirmation** |
| **RSP2_R27_3xP2down** | CTGGCTCTGCATTCGAATTTC | **RSP2-Flag confirmation** |
| **R27_RSP2_pLG EcoRI fw** | CGGAATTCCTACATCTGCGTCGTACTGAA | ***rsp2* cloning** |
| **R27_RSP2_pLG BamHI rv** | CGGGATCCTGCGACTAAATCAGCCTGTTT | ***rsp2* cloning** |
| **trhH_R27_P1** | ACTATCTGAATGTGGGTGGAAGTTTAGGAGGTGCATATGCGCGTGTAGGCTGGAGCTGCTTC | ***trhH* deletion** |
| **trhH_R27_P2** | CGCCAACTGTATAGATGTTGTAATCCATATCAGACCTCAGTTCATATGAATATCCTCCTTAGT | ***trhH* deletion** |
| **trhH_R27_P1up** | ACCATTGGCGAGGATGGCGTA | ***trhH* deletion confirmation** |
| **trhH_R27_P2down** | GGCTGGCTCCAGCCAGTACCG | ***trhH* deletion confirmation** |
| **trhA_R27_P1** | TGCGTTTTCGTGAATTCAAATCAACACGGAGTAATTATGGAAGTGTAGGCTGGAGCTGCTTC | ***trhA* deletion** |
| **trhA_R27_P2** | GGGGAATATCCCCCTCATTGTTATTGTTCTAACAAATCACAGCATATGAATATCCTCCTTAGT | ***trhA* deletion** |
| **trhA_R27_P1up** | GGATGAATGCCATAAAAATGG | ***trhA* deletion confirmation** |
| **trhA_R27_P2down** | CCGCTCGCGATAGTCACGAAT | ***trhA* deletion confirmation** |
| **KT** | CGGCCACAGTCGATGAATCC | **Confirmation Km^R^ insertion** |
| **CatC1** | TTATACGCAAGGCGACAAGG | **Confirmation Cm^R^  insertion** |
| **PkmXmnIFw** | GAAACGTTTCAGCACTCAGGGCGCAAGGGCT | **Amplification Km^r^ for cloning into pKD46** |
| **PkmXmnIRv** | GAAACGTTTCTCAGAAGAACTCGTCAAGAAG | **Amplification Km^r^ for cloning into pKD46** |
| **ALG87338.1P1** | TTGGGTTCTTGTAACTCAAAACGAATGGAGAATGCGATGATCGTGTAGGCTGGAGCTGCTTC | ***ALG87338.1* deletion** |
| **ALG87338.1P2** | ATTGCGGTCTGAACCGGGCTTTGAGAGGAATGAACCTTAGTTCATATGAATATCCTCCTTAGT3 | ***ALG87338.1* deletion** |
| **ALG87338.1P1up** | ACGGGTGATAGGGGCAGCCTA | ***ALG87338.1* deletion confirmation** |
| **ALG87338.1P2down** | ATGCCATCACACTTCGCAGC | ***ALG87338.1* deletion confirmation** |
